# Investigating a propagation of emerging carbapenemase-producing Enterobacteriaceae in Dutch broiler production pyramid through stochastic simulation

**DOI:** 10.1016/j.onehlt.2024.100945

**Published:** 2024-11-26

**Authors:** N. Dankittipong, J.A. Stegeman, C.J. de Vos, J.A. Wagenaar, E.A.J. Fischer

**Affiliations:** a Faculty of Veterinary Medicine, Utrecht University, Yalelaan 7, Utrecht, the Netherlands; b Wageningen Bioveterinary Research, Wageningen University & Research, Houtribweg 39, Lelystad, the Netherlands

**Keywords:** Simulation model, Broiler production, Antibiotic resistant bacteria, SimInf, Transmission dynamics model, Carbapenem-resistant bacteria, Emerging resistant bacteria

## Abstract

Simulating resistant bacteria transmission in livestock informs surveillance strategies for emerging threats like Carbapenem-resistant Enterobacteriaceae (CPE), aiding targeted surveillance and detecting CPE through active methods. We employed a simulation model to assess three potential scenarios for introducing CPE: 1) a single import of live animals, 2) the use of contaminated feed, and 3) multiple imports of live animals. Employing the SimInf package, we constructed a population model for broiler production, encompassing rearing farms, multiplier farms, hatcheries, and broiler farms. Subsequently, we introduced CPE and allowed it to spread throughout the population using the Susceptible-Colonized (Infectious)-Susceptible model. The model ran for 10 years with 100 runs.

In the single import scenario, 1–2 rearing and multiplier farms saw major outbreaks in all 100 runs, while the broiler farm experienced major outbreaks in only 10 out of 100 runs; in the feed scenario, major outbreaks occurred in rearing farms in 32 runs and in multiplier farms in 26 runs, with major outbreaks in broiler farms observed in all 100 runs; in the multiple import scenario, outbreaks in rearing and multiplier farms happened in all 100 runs, with these major outbreaks reaching the broiler farm in 91 out of 100 runs. CPE transmission from imported or colonized broilers is rapid but short-lived within the broiler population, contrasting with the sporadic and prolonged emergence of CPE from contaminated feed, resulting in lower cumulative probabilities of detection from imported or colonized animals (0–0.50) compared to contaminated feed (0.9–0.97) over a 10-year period. Sensitivity analysis indicated that key outcomes such as farm outbreaks, chicken colonization, and outbreak duration are highly correlated with age-associated reductions in transmission (ψ).

## Highlights

• Probability of detecting CPE is higher in feed introduction.
• Reduced susceptibility to colonization is the key parameter affecting the spread.
• CPE spreads immediately after introduction, but detection depends on the type of farm introduced.

## 1 Introduction

Carbapenem-resistant Enterobacteriaceae (CPE) pose a significant threat to public health due to their ability to resist the clinically important carbapenems. CPE emergence is particularly concerning because carbapenem-resistance genes are frequently located on plasmids, which are mobile genetic elements that can rapidly transfer between bacteria and facilitate the transmission of resistance between humans and animals [1,2]. Furthermore, CPE can arise from the use of other commonly administered antibiotics through co-resistance [3,4].

Despite the prohibition of carbapenem use in livestock, on a global scale, the emergence of CPE in wildlife, companion animals, and livestock has been observed since 2009 [1,5]. This emergence raises concerns about the potential spillover of CPE into the livestock population in the Netherlands, which, in turn, could serve as a source of introduction to the human community through the consumption of CPE-contaminated meat [2,6].

In recent decades extended-spectrum beta-lactamase (ESBL) producing bacteria have risen to high levels in Dutch poultry production. Although these levels have dropped considerably following a strong reduction in antibiotic use, this example shows the risk of plasmid-mediated resistance [7]. Other plasmid-mediated resistant bacteria are also prevalent in broilers. CPE is plasmid-mediated and exhibits co-resistance with some of these resistant bacteria. This makes poultry a crucial target for surveillance efforts [8].

Considering their significance in antimicrobial resistance (AMR), broiler production is prioritized for surveillance activities [4,8,9]. The current national surveillance in the Netherlands tests a small proportion of the animal population in the slaughterhouse per year [10]. This protocol may not effectively detect emerging AMR at an early stage when curtailing its spread would still be feasible. For example, the detection of extended-spectrum beta-lactamases (ESBLs) in poultry occurred only after they had become widespread [11]. Additionally, AMR may be introduced in farm types that are not included in current surveillance programs. This leaves a considerable proportion of the animal population exposed to colonization by newly emerging AMR strains before their presence has been discovered.

To gain a comprehensive understanding of the spread and persistence of CPE throughout the entire broiler production pyramid, it is imperative to systematically integrate the available information on CPE transmission dynamics. Meta-population models can be used to simulate the population dynamics of colonization within herds and between interconnected herds as has been demonstrated for exploring the transmission of bacterial diseases between broiler and pig farms [[12], [13], [14]]. Such models can be useful to investigate population dynamics of CPE, but also to evaluate surveillance [[15], [16], [17]].

We aim to quantify the consequences of CPE introduction into the Dutch broiler production chain. Our main outcomes will be the number of farms experiencing an outbreak of CPE, the duration of the outbreaks, the number of contaminated batches and birds at slaughter, and the probability of detection with the current surveillance system. We investigate the two potentially most important routes of introduction, namely the import of live birds and contaminated feed [18].

## 2 Materials and methods

### 2.1 Simulation model

We adapted an epidemiological simulation model for antibiotic resistance transmission within and between farms for a part of the poultry production pyramid [12]. The model tracks flocks of broiler chickens and parent stock chickens (referred to as PS chickens) instead of farms, as farms may house multiple flocks of broiler chickens and PS chickens annually. We parameterized the model for CPE. In addition, we included the current Dutch CPE surveillance system in the model and performed a sensitivity and what-if analysis. We used a stochastic discrete-time simulation model in which the spread of CPE and population dynamics of rearing, multiplier farms, hatcheries, and fattening farms are explicitly simulated. The SimInf package for R was used to implement the model. The initial number of PS chickens raised in rearing farms, obtained from Statistics Netherlands, served as the starting point (Statistik, 2023). After each production round, the PS chickens were moved to multiplier farms. From there, the eggs moved through the hatchery, resulting in hatched broiler chickens that were subsequently sent to broiler farms.

The transmission dynamics were simulated for a period of 10 years (3650 days) in each run, with a total of 100 iterations generated to assess the spread of CPE resulting from feed contamination and the import of live animals.

### 2.2 Epidemiological model

#### 2.2.1 Compartmental model and transition within compartment

The course of within-flock outbreaks of CPE was modeled with a stochastic Susceptible-colonized-Susceptible (SIS) compartmental model with environmental transmission (Table 1). The susceptible animals (S) are colonized by CPE in the environment at a colonization coefficient of CPE (β). This SIS compartment model was chosen based on the observed status of CPE-colonized broiler chickens in transmission experiments, where broilers lost colonization and became recolonized [20]. The colonized animals (I) can recover and become susceptible animals$\;\left(S\right)$ at a rate of recovery (γ). Please note that to be consistent with literature on SIR models, we use the symbol *I* for colonized animals, although CPE is not an infection.

**Table 1:** Transition states of the within-flock SIS compartmental model with environmental transmission and age-dependent transmission reduction [12]. $\psi \left(a\right)\;$= decrease of susceptibility with age (days); $\varphi \left(t\right)$ is the environmental contamination at time t. β is colonization coefficient; ω is the colonization rate through feed; type of bird k = ps (parent stock) or b (broiler); γ is recovery rate; l is rate of pseudo vertical transmission; b is egg laying rate, ν is probability of contamination of egg by PS chickens; subscript m is multiplier bird; h is hatching rate.

Health state transition		Rate
S→I	Colonization from susceptible parent stock or broiler bird (*S*) to colonized parent stock or broiler bird (*I*)	($z-\left(z+1\right)∙e^{-\psi \left(a\right)})∙\left(\varphi \left(t\right)\beta +\omega_{k}\right)$
I→S	Recovery from colonized (*I*) to susceptible (*S*)	γ
→EE	Production of uncontaminated eggs (*EE*)	$b\left(S_{m}+\left(1-l\right)I_{m}\right)$
→EI	Production of contaminated eggs (*EI*)	$b∙I_{m}∙\nu$
EE→S	Hatching of susceptible chickens from uncontaminated eggs (*EE*)	h∙EE
EI→S	Hatching of susceptible chickens from contaminated eggs (*EI*)	$h\;\left(1-\nu \right)∙\mathrm{EI}$
EI→I	Hatching of colonized chickens from contaminated eggs	h∙ν∙EI

Introduction of CPE occurs either by a constant rate of CPE colonization from feed (ω) specific for both parent (subscript ps) and broiler chickens (subscript b) or by the import of colonized chicks. After introduction, CPE spreads via the environment between chickens at time t at rate given by the contamination ($\varphi \left(t\right)\;$) and colonization coefficient of CPE (β). We assume an age-dependent decreasing susceptibility of chickens ($e^{-\psi \left(a\right)})$ mimicking the maturation of the gut microbiome (Table 2). The age-dependent susceptibility was fitted on observational data from broilers with only a short time span leading to resistance at higher age of parent stock [[21], [22], [23]]. An alternative model, in which a minimum susceptibility $\left(z\right)$ was assumed, was included such that the age-dependent susceptibility was $z-\left(z+1\right)\;e^{-\psi \left(t\right)}$.

**Table 2:** Input parameters for the CPE SIS model in broiler chickens.

Values	Parameter description	Values (unit)	References
b(t)	The daily eggs laying rate by PS chickens in multiplier farm	0.12 (day^−1^)	
ν	The probability of contamination of egg by PS chickens	0.004	[25]
*l*	The pseudo vertical transmission rate to from hatchery environment to eggshell	0.0036 (day^−1^)	[25]
β	Colonization coefficient of CPE	0.0048 (day^−1^)	[20]
h(t)	The daily hatching rate in the hatchery	0.05 (day^−1^)	[26]
γ	The recovery rate	0.03 (day^−1^)	[27]
$\varphi \left(t\right)$	The rate of colonization from feces in the environment		
$p_{\mathrm{fee}d_{\mathrm{ps}}}$	The probability of colonization from feed to parent flock	0.05 (year^−1^)	[18]
$p_{\mathrm{fee}d_{b}}$	The probability of colonization from feed to broiler flock	0.23 (year^−1^)	[18]
$\omega_{\mathrm{ps}}$	The rate of colonization from CPE-contaminated feed to parent flock	$1.4∙10^{-8}$(day^−1^)	
$\omega_{b}$	The rate of colonization from CPE-contaminated feed to broiler flock	$7.0∙10^{-8}$(day^−1^)	[27]
θ	The rate of CPE excretion into the environment of animal's pen	2.70 (day^−1^)	[27]
$N_{\mathrm{anima}l_{\mathrm{broiler}}}$	The average number of broiler chickens in a broiler farm	10,000	
$N_{\mathrm{anima}l_{\mathrm{PS}}}$	The average number of PS chickens in a rearing and multiplier farm	40,000	
ρ	The survival rate of excreted CPE	0.62 (day^−1^)	Fitted to data of [22]
ψ	Reduction in the probability of colonization with time (as chickens age)	0.6 (day^−1^)	[[21], [22], [23]] Fitted to data of [22]
z	The minimum susceptibility	0.10	Fitted to data of [22]

Contamination in the hatchery comes from the eggs produced by colonized parent broilers (EI). CPE-contaminated eggs are calculated from the number of eggs produced by colonized PS chickens multiplied with the probability of contamination of eggs by PS chickens(ν).

#### 2.2.2 Transmission parameters

The rate of colonization from CPE-contaminated feed was calculated from the annual probability of colonization of a flock due to CPE contamination in feed ($p_{\mathrm{feed}}$) estimated in a previous study [18]. We calculate the daily rate of introduction per animal as follows:

(1) $$\omega =-\frac{\ln\left(1-p_{\mathrm{feed}}\right)}{365∙N_{\mathrm{animal}}}\;$$

To determine the rate of colonization from feces in the environment ($\varphi \left(t\right)$), we modeled the excretion of viable CPE from colonized animals. We assumed that a colonized animal (I) at time *t* excretes CPE into the environment of its pen at a constant rate of $\left(\theta \right)$ units per day. The excreted CPE remains viable for transmission according to a survival rate of $\left(\rho \right)$ per day (eq.2).

(2) $$\varphi \left(t\right)=\rho \;\varphi \left(t-1\right)+\theta \cdot I$$

We used the colonization coefficient of CPE (β) estimated from a transmission experiment of CPE in broiler chickens [20].

Our model includes that animals become less susceptible to colonization as they age [[21], [22], [23], [24]]. Our baseline model assumes an exponential decrease of susceptibility with a reduction rate (a), which is fitted on data from a broiler flock. This would lead to approximately zero susceptibility in older parent birds, which is not in agreement with the abovementioned studies. Therefore, an alternative model with a minimum susceptibility (z) was also simulated. The transmission reduction (*ψ(a)*) and a minimum susceptibility (*z*) were fitted according to the data of Huijbers et al. (2016) using Approximate Bayesian Computation Sequential Monte Carlo (ABC-SMC) [12].

### 2.3 Population dynamics

#### 2.3.1 Farm structure, size, and type

**Fig. 1:**
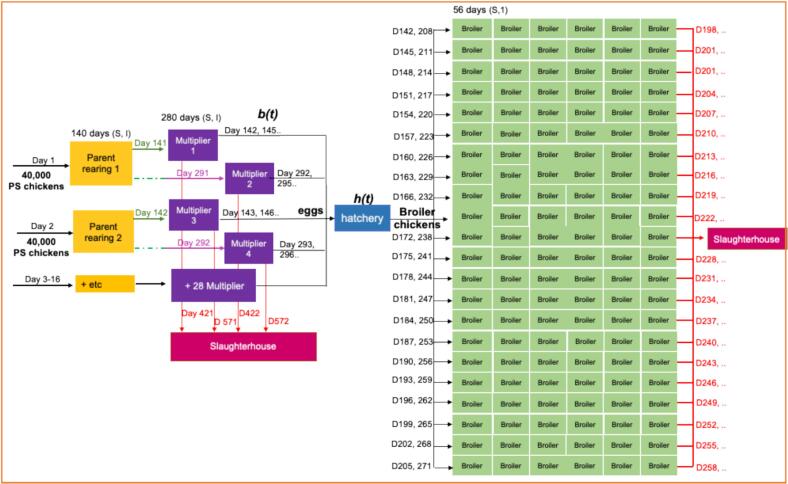
Farm structures in the broiler production. Arrows represent the flow of parent broiler chickens (PS chickens), eggs, and broiler chickens. Day and D indicate the day the movement occurs. b(t) is the parent broiler laying rate per day and h(t) is the egg hatching rate per day. The simulation covers a period of 4000 days and begins with 40,000 PS (parent stock) chickens entering the rearing farms (yellow-colored boxes). These chickens are then transported to multiplier farms (purple-colored boxes). Eggs are transferred to the hatchery (blue-colored box) where they hatch. The resulting one-day-old broiler chickens are transported to broiler farms (green-colored boxes). Red-colored arrows indicate the movement of PS chickens and broiler chickens to slaughterhouses. (For interpretation of the references to colour in this figure legend, the reader is referred to the web version of this article.)

The four farm types most important for the Dutch broiler production sector are considered: rearing farm (*n* = 90), multiplier farm (*n* = 200), hatchery (*n* = 6), and broiler farm (*n* = 780). All rearing farms and multiplier farms were assumed to house 40,000 animals. The numbers of farms were based on a publicly available national database, Statistics Netherlands (CBS). The movements of animals within the production are based on experts in broiler rearing [19]. The queries to retrieve the data are detailed in Supplementary Material 2. We simulated the movements of parent broiler chickens (PS chickens), eggs, and broiler chickens across a network of 181 farms. This enabled us to simulate the boiler production sector with the smallest number of farms, including all farm types in the production chain (Fig. 1), which facilitated the computations. In the simulation, the numbers of farms included are rearing farm (*n* = 16), multiplier farm (*n* = 32), hatchery (n = 1), and broiler farm (*n* = 132). This selection was made because our primary interest was in single introductions and feed that would be present in all farm types except hatchery, and therefore, we focused on simulating the network around a single hatchery. We did not simulate all the farms in the broiler production sector because we assumed that the connections between rearing farms, multiplier farms, hatchery, and broiler farms form a closed network. As such, we assumed that the rest of the farms in the broiler production sector would exhibit the same closed network properties as this group of 181 farms. Additionally, for efficiency and expediency, we chose to concentrate our efforts on this unit of a closed network rather than simulating the entire country, as the outcomes would be analogous but on a larger scale.

#### 2.3.2 Production round

According to the production procedure, we simulated all-in-all-out production rounds in parent-rearing farms, parent multiplier farms, and broiler farms. Hatcheries are modeled as a continuous flow system. Transport and production are calibrated so that the numbers of animals, farms, and hatcheries match.

Parent-rearing farms are stocked every 150 days with 40,000 one-day-old parent broiler chickens (PS: Parent stock) that are raised for 140 days. After 140 days, these PS chickens are transported to one of the two multiplier farms connected to this farm (Fig. 1). After a downtime of 10 days, the parent-rearing farm will receive a new batch of one-day-old PS chickens.

In the multiplier farm, PS chickens produce eggs at a rate of *b(t)* per day that are transported to the hatchery. PS chickens are kept in a multiplier farm for a period of 280 days, after which they will be slaughtered at the age of 420 days (140 days in the rearing farm and 280 days in a multiplier farm) [28](Table 2). The 32 multiplier farms are transporting their eggs to a single hatchery on alternate days. In the hatchery, eggs are hatched at the rate of *h(t)* per day.

The number of eggs in the hatchery and chicks in broiler farms will be subject to a stochastic process characterized by variations in hatching and laying rates in PS chickens (Table 2).

After hatching, one-day-old broiler chickens are transported to broiler farms. 3000 to 89,000 one-day-old chickens are transferred to each of 6 different farms every 3 days. To illustrate, on Day 0, broiler chickens are delivered to broiler farms 1 to 6, while on Day 3, broiler farms 7 to 12 receive the broiler chickens, etc. After 66 days (completing 22 transport rounds), the hatchery will restart the transportation process to the same broiler farms, repeating the round. Slow-growing broiler chickens are reared in the Netherlands for 56 days.

The parameters dictating the moment of transition between the farms is collated in supplementary data (Table S1).

### 2.4 Probability of detection by slaughterhouse monitoring

Based on the current national surveillance protocol for CPE in broiler production [7] and the simulated number of contaminated and slaughtered chickens, we calculated the probability of detecting CPE in a batch of broiler chickens sent to slaughterhouses (${P_{\det}}_{i})$ (Table 3). The probability is calculated by multiplying the probability that a batch of broiler was sampled during slaughterhouse surveillance with the probability that the batch of broiler *i* has at least one colonized broiler chick and would test positive for CPE if it is indeed colonized ($P_{\mathrm{posde}t_{i}})$:

(3) $$\;{P_{\det}}_{i}=\frac{{\mathrm{sample}}_{\mathrm{NL}}}{N_{\mathrm{broilerfarm}}∙N_{\mathrm{broilerbatch}}}∙\left(1-{\left(1-\frac{I_{s}}{N_{s}}\mathrm{Se}\right)}^{n}\right)$$

**Table 3:** Input to calculate the probability of detection CPE at slaughter.

Input	Description	Value (unit)	Reference
${\mathrm{sample}}_{\mathrm{NL}}$	Total number of broiler batches sampled per year in the national surveillance; each flock sampled came from a different broiler farm	300 (year^−1^)	[7,30]
$N_{\mathrm{broilerfarm}}$	Total number of broiler farms in the Netherlands	780	AVINED expert; [19]
$N_{\mathrm{broilerbatch}}$	Total number of flocks of broilers raised in a broiler farm per year	5.5 (year^−1^)	[28]AVINED expert)
$\frac{I_{s}}{N_{s}}$	Proportion of CPE-positive broiler chicken at slaughter age	proportions	Obtained from simulations
Se	CPE test sensitivity	0.85	[29]
n	Number of samples per flocks for CPE surveillance	10 (flocks)	[7,30]; Expert

The probability that a flock of broilers was sampled during annual surveillance is calculated by dividing the total number of broiler batches sampled annually (${\mathrm{sample}}_{\mathrm{NL}}$) with the number of broiler batches raised per year. The probability that at least one broiler chicken in a flock of broiler chickens *i* is CPE positive and is sampled in the surveillance ($P_{\mathrm{posde}t_{i}})$ is the fraction of broiler chickens in a batch being colonized by CPE at slaughter ($\frac{I_{s}}{N_{s}}$) and test sensitivity $\left(\mathrm{Se}\right)$. $P_{\mathrm{posde}t_{i}}$ varies depending on the route of introduction because it depends on the number of colonized broiler chickens at slaughter. Test sensitivity $\left(\mathrm{Se}\right)$ is based on the CPE screening report conducted by National Institute for Public Health and the Environment and *n* is the number of samples per farm [19,29]. We assume a 100 % specific test.

The number of samples per flock in the national surveillance was set to 10 samples per flock, following the sampling protocol outlined by the national surveillance for resistant bacteria in livestock (Expert).

### 2.5 Outcomes

We evaluated the spread of CPE through five outcomes: (1) the number of farms that have a major outbreak of CPE (N_farm_), (2) the number of animals colonized with CPE (N_col._), (3) the duration of CPE colonization on flocks (D), (4) the reoccurrences of outbreaks in the same farm, and (5) the number of colonized animals sent to the slaughterhouse over the simulation period. For a farm to be classified as having a major CPE outbreak, the cumulative number of colonized birds must exceed 80. It should be noted that a major outbreak in a hatchery involves at least one contaminated egg, given that eggs are continually hatched and transferred to broiler farms.

#### 2.5.1 Introduction of CPE scenarios

We investigated the spread of CPE through the introductions from feed and import of live animals, which are the most likely sources of CPE introduction to broilers in the Netherlands [18]. Three introduction scenarios were simulated. In the first introduction model, we simulated introductions by colonized PS chickens in a single flock of import into a rearing farm (single import model), to model the consequences of rare introductions. To minimize the risk of stochastic fade out, we assumed each batch would include 40 CPE-colonized birds. For feed, a continuous exposure of CPE in rearing, multiplier, and broiler farms was simulated (feed model) according to the potential exposure estimated from the risk assessment. Lastly, the introduction of 20 colonized PS chickens in every flock of a rearing farm (multiple import model) was simulated to mimic the impact of a constant influx of colonized chickens. In the model, CPE was introduced after a one-year (warm-up) simulating period to the stable number in the population.

#### 2.5.2 Sensitivity analysis

Sensitivity analysis was used to assess the robustness of the model prediction to changes in model input and structure [31]. In the sensitivity analysis, we changed parameters one-at-a-time with a factor 0.1 and 2 (Table 4). All parameters were evaluated. We applied the sensitivity analysis to two models: with introduction from feed and with single introduction from import. The number of runs in the sensitivity analysis is 10 for each parameter combination. The key output variables used to assess the influence of each parameter included the total number of positive flocks, the total number of positive animals, the duration of the period of contagiousness, and the number of CPE-colonized broiler chickens at the slaughterhouse.

**Table 4:** Parameters included in the sensitivity analysis. Parameter values displayed 10 % of the original value, original value, and a 100 % increase of the original value.

Parameter changes	Farms	Parameter	Parameter values
Rate of exposure to CPE from feed	Rearing farm, Multiplier farm	$\omega_{\mathrm{ps}}$	$1.4∙10^{-10},1.4∙10^{-8},2.8\cdot 10^{-8}$
	Broiler farm	$\omega_{b}$	$7∙10^{-10},7∙10^{-8},1.4\cdot 10^{-7}$
Proportion of colonized PS chicken in a batch of import	Rearing farm	Proportion of colonized PS chickens in one batch of import	0.0001, 0.001 (40 PS chickens), 0.002 (80 PS chickens)
Recovery rate	Rearing farm, Multiplier farm	γ	0.003, 0.03, 0.06
	Broiler farm		
Reduction in susceptibility to transmission due to age	Rearing farm, Multiplier farm, Broiler farm	ψ	0.006, 0.6, 1.2
Minimum susceptibility	Rearing farm, Multiplier farm, Broiler farm	z	0.01, 0.10, 0.20
*E. coli* carrying CPE shedding rate	Rearing farm, Multiplier farm, Broiler farm	θ	0.005, 0.5, 1
*E. coli* carrying CPE survival rate in the environment	Rearing farm, Multiplier farm, Broiler farm	ρ	0.005, 0.05, 0.1
Time-dependent environmental transmission rate	Rearing farm, Multiplier farm, Broiler farm	β	0.00048, 0.0048, 0.0096

#### 2.5.3 What-if analysis

In what-if analysis, we explored six different scenarios in broiler production, considering variations in farm characteristics, connections, and exposure to the risk of CPE (Table 5). Scenarios 1 to 6 were implemented for a model with introduction from single import and, additionally, Scenarios 5 and 7 were implemented for introduction by feed.

**Table 5:** The changes made to the simulation model to investigate possible scenarios in the broiler production process.

Scenario	Descriptions	Farm types adjusted	Model
Baseline	Single import model	All rearing farms	40 colonized PS chickens in one batch Zerocolonization rate from CPE-contaminated feed in parent flock Slaughter age = 56 days
	Feed model	All farms	Zero colonized PS chickens in one batch $1.4∙10^{-8}\;$colonization rate from CPE-contaminated feed in parent flock $7.0∙10^{-8}$ colonization rate from CPE-contaminated feed in broiler flock Slaughter age = 56 days
1	All rearing farms import PS chickens from outside of the Netherlands	All rearing farms	20 of colonized PS chickens imported to 16 rearing farms in every batches
2	All rearing farms import PS chickens from outside of the Netherlands half of the time	All rearing farms	40 of colonized PS chickens imported to 16 rearing farms in every other batches
3	Broiler farms imported additional batch of broiler chickens from hatchery outside the Netherlands once per year	All broiler farms	20 colonized broilers out of 20,000 broiler chickens are imported from outside NL to 132 broiler farms once per year
4	Broiler farms imported additional batch of broiler chickens from hatchery outside the Netherlands in every round	All broiler farms	4 colonized broilers out of 20,000 broiler chickens are imported from outside NL to 132 broiler farms 5.5 times per year
5	The lifespan of chicken is shorter reflecting the slaughter age of conventional broiler chicken	All broiler farms	The slaughter age is at 42 days old
6	Broiler chickens in all broiler farms receive antibiotics.	all rearing farms, all multiplier farms, all broiler farms	The transmission rate of CPE increases to 0.048 in all farms
7	Localize the exposure to feed to 10 % of broiler farms.	Broiler farms	The colonization rate from CPE-contaminated feed in broiler flock ($\omega_{b})$ is $7∙10^{-8}$ in 13 broiler farms across the length of simulation, whereas 119 broiler farms have zero rate of exposure throughout the length of simulation

## 3 Results

### 3.1 Introduction of CPE scenarios

As stated above, three introduction models were simulated: 1) single import of live animals, 2) use of contaminated feed (feed), and 3) multiple imports of live animals. The introduction from import was assumed in a single rearing farm, either once (single import) or multiple times in the same farm (multiple imports). The introduction of CPE into the broiler production pyramid from feed was assumed to be continuous in all farms. In the case of the hatchery, where there is a continuous flow of eggs and animals every two days, contamination was considered present if at least one positive egg is present. We compiled the median, 5th percentile, and 95th percentile of all outcomes in Table 6.

**Table 6:** Outcome of three introduction models; 1) Single import of colonized PS chicken into a rearing farm, 2) multiple import of colonized PS chicken into the same rearing farm, and 3) contaminated feed in rearing, multiplier, and broiler farms.

Introduction models⁎		Single import	Multiple import	Feed
N_farm_	Rearing	1 [1,1]	1 [1, 1]	0 [0,2]
	Multiplier	1 [1, 1]	2 [2,2]	0 [0, 2]
	Broiler	0 [0, 6]	13 [1, 25]	56 [45, 64]
N_col_	PS chickens in rearing	39,932 [39,919, 39,945]	1,077,590 [1,077,478, 1,077,712]	0 [0,1323]
	PS chickens in multiplier	793 [753, 836]	20,581 [20,362, 20,797]	0 [0, 63,867]
	Eggs in hatchery	10 [6, 15]	275 [249, 297]	0 [0, 16]
	Broiler chickens in broiler farms	0 [0, 390,269]	860,365 [63,096, 1,853,520]	4,209,142 [3,338,773, 5,333,092]
D	PS chickens in rearing	138 [138, 138]	138 [99, 138]	136 [133, 137]
	PS chickens in multiplier	236 [184, 278]	228 [166, 279]	198 [63, 261]
	Broiler chickens	54 [54, 54]	54 [54, 54]	52 [48, 54]
Prevalence at slaughter in colonized broiler	Broiler chickens	0.26 [0.25, 0.26]	0.26 [0.25, 0.26]	0.25 [<0.01, 0.26]
N_farm_ at slaughter	Broiler chickens	0 [0, 6]	13 [1, 25]	63 [53, 71]

⁎ N_farm_ is number of farms with a major outbreak, N_col_ is number of colonized chickens OR number of contaminated eggs, D = duration of outbreak within a farm.

#### 3.1.1 Major outbreaks in the simulations

**Fig. 2:**
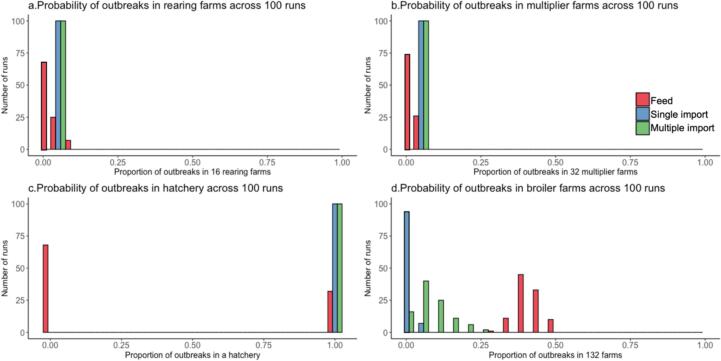
Probability distribution of the proportion of farms experiencing a major outbreak and contamination (hatchery) across three introduction scenarios, feed, single import, and multiple imports. The simulations contain 16 rearing farms (a), 32 multiplier farms (b), 1 hatchery (c), and 132 broiler farms (d). The X-axis indicates the proportion of farms that experience major outbreaks and contamination of the hatchery. The total number of runs with the outbreak is on the Y-axis.

When a rearing farm received a batch of 40 contagious PS chickens (single import), all runs resulted in a major outbreak in the rearing farm and the connected multiplier farms was always colonized. The presence of colonized PS chickens in the multiplier farms led to the production of a small number of contaminated eggs in all 100 runs. Despite the presence of contaminated eggs, the number of day-old colonized broiler chickens remains low in the hatchery due to the relatively small quantity of contaminated eggs and low probability of colonization of the chicks from the eggs. However, this small number of contaminated eggs can lead to secondary transmission in the broiler farms. Contrary to other farm types, a small number of contaminated eggs of at least one is categorized as a major outbreak in Fig. 2 as the eggs are moved from hatchery to broiler farms every two days, thus, the number of contaminated eggs would not accumulate to be as high as 80 contaminated eggs. The hatched eggs resulted in major outbreaks in broiler farms in 10 out of 100 runs, with the number of affected farms varying between 1 and 6 (Fig. 2). Repeated outbreaks in the same farm were not observed in this scenario.

**Fig. 3:**
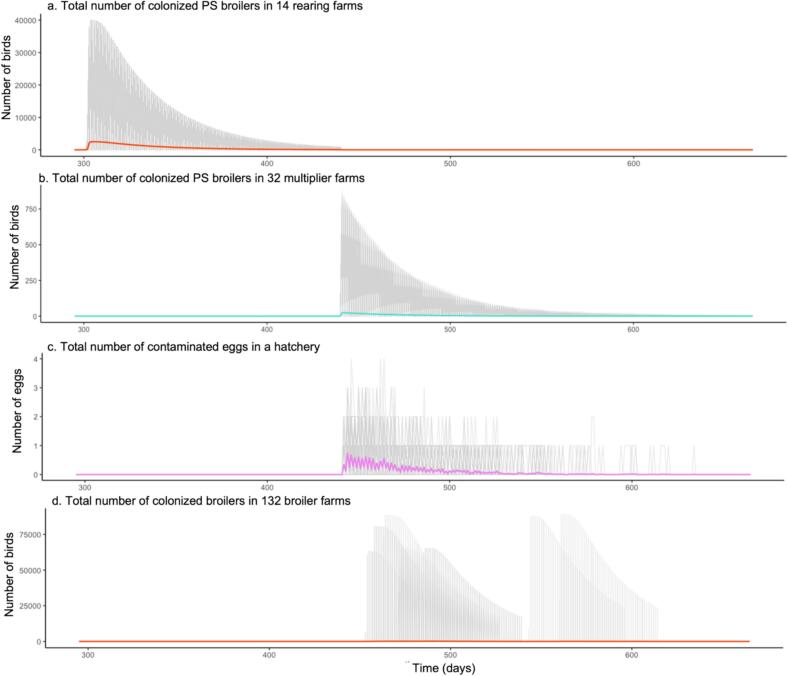
The average number of animals colonized with CPE introduced from a single import across all farms from 100 runs. Each plot shows the number of animals in each farm type from Day 300 to Day 665. The gray lines represent the number of colonized animals from individual runs (100 in total), while the colour-coded lines indicate the average number of animals colonized within each farm type.

In the scenario of multiple import introductions, where 20 colonized PS chickens were introduced with each batch to the one specific rearing farm, it was observed that the rearing farm became colonized in all runs as well as the two multiplier farms connected to this rearing farm and their accompanying hatchery. Repeated outbreaks were observed in the rearing farm, aligned with the repeated introductions of colonized PS stock. In between introductions, the contamination faded out (Fig. 3). In this scenario, major outbreaks in broilers occurred in 91 out of 100 runs with the number of colonized farms varying between 8 and 24 (Fig. 2). In addition, repeated outbreaks occurred within the same broiler farm in 13 out of these runs, with the number of outbreaks ranging from 1 to 6. Most of the time, the hatchery received one contaminated egg per day, while for less than 10 % of the time, they received 2 to 4 contaminated eggs. We regard at least one contaminated egg as a major outbreak. This pattern was consistent across all runs, indicating that the instances of recurrence were random chance events. In the simulation, each broiler farm supplied a total of 51 batches of broiler chickens to the slaughterhouse. Consequently, the majority of farms delivered a batch with colonized animals.

Continuous exposure from feed resulted in major outbreaks in rearing farms in 32 runs and in multipliers in 26 runs. Major outbreaks in multiplier farms resulted in a major outbreak of at least one contaminated egg in a hatchery in 23 runs. In both farm types, the number of affected farms varied between 1 and 2. Recurring series of outbreaks within the same farm were observed in 10 of the runs in rearing farms, but not in the multiplier farms. All runs experienced series of major outbreaks in broiler farms, with the number of affected farms varying between 48 and 54. Repeated outbreaks in the same broiler farm occurred in all 100 runs, with most of these being limited to one repeated outbreak affecting 2 to 18 farms, while 5 farms experienced two repeated outbreaks. Rarely, one farm would have 3 recurring outbreaks. Broiler farms exhibited an almost five-fold higher probability of colonization per year compared to other farm types (Table 4). This elevated probability is attributed to several factors, including a higher number of batches per year, younger chickens that are more susceptible to colonization, and the resulting increased likelihood of introduction through contaminated feed.

#### 3.1.2 Number of chickens colonized with CPE

The transmission dynamics of CPE spread in rearing farms and broiler farms follow a similar pattern of rapid spread, affecting 99 % of the flock within 1 to 3 days after introduction, and subsequently declining gradually, irrespective of the source of introduction. Both rearing and broiler chickens are relatively young (1 to 8 days) and are thus, very susceptible to colonization.

On a rearing farm, when CPE is introduced through 40 colonized rearing chickens, the number of colonized PS chickens rapidly peaks with number of 39,992 colonized chickens (99 % of the PS chickens in the farm) after 3 days (Fig. 3a). Afterward, the number of colonized PS chickens slowly declines due to recovery and a decreasing susceptibility with age. It reaches 800 to 900 chickens at the time of transport to the multiplier farm.

Next, the number of colonized PS chickens on the multiplier farm gradually declines from 800 to 400 due to further increasing age resistance or recovery (Fig. 3b). Ultimately, the multiplier farm becomes free of CPE after 210 days (when the PS chickens are approximately 350 days old). According to the simulations, these colonized PS chickens produce only 6 to 15 contaminated eggs in the entire round (Fig. 3c). Consequently, outbreaks in broiler farms are rare, occurring in only 10 out of 100 runs (as shown in Fig. 2). However, when such outbreaks do occur, they spread rapidly and reach a maximum of colonized broiler chickens after 1 to 4 days throughout the entire broiler farm population, affecting between 60,000 to 88,000 chickens (99 % of the chicken in a batch) (Fig. 3d).

The introduction of CPE through multiple imports leads to similar dynamics within each of the farm types, although the occurrence of the outbreaks is more frequent due to repeated introductions into the rearing farm. Although the quantity of contaminated eggs increases to 22 to 248 eggs in this scenario, the number of colonized broiler farms remains relatively low (Fig. 2c).

Contaminated feed results in more variability in the size of an outbreak in rearing farms and broiler farms than single or multiple import. In rearing farms, the peak of spread from feed introduction ranges from 20,000 to 29,492 chickens (50 to 70 % of the population), which represents the 5th to 95th percentile of the population. On rare occasions, the peak can reach between 100 and 39,207 chickens (0.25 % to 98.0 % of the chickens in the batch). The time to reach the peak varies from 7 to 14 days, where a longer time until the peak results in a lower peak, due to the age-dependent susceptibility (ψ) assigned in all introduction models. A similar variation is observed in broiler farms, where the percentage of colonized animals ranges from 0.1 % to 99 %, and the time to peak ranges from 5 to 11 days. Importantly, in the feed scenario, only very young animals can become colonized in both broiler and rearing farms. Moreover, if the introduction of contaminated feed occurs on Day 8, the probability of a major outbreak is small, as only 0.2 % of animals are colonized by that age. Overall, the dynamics of spread in the explored introduction scenarios exhibit variations but are all capable of causing major outbreaks in the flocks.

After the outbreak peaks, typically affecting 80–99 % of the flock, there is a rapid surge in the number of colonized chickens, followed by a steady decline. This decline is consistent across all introduction routes due to the assumption of age-dependent susceptibility, which reduces the number of colonized animals as the production cycle progresses, thereby reducing CPE contamination in the farm environment. Following each production round, the entire flock of birds is relocated to a different farm, resulting in an average downtime of 10 days. During this downtime, the remaining CPE colonization in the environment, which has already been reduced due to the lower number of colonized animals, continues to decrease. Additionally, cleaning and disinfection procedures carried out during this downtime further eliminate any remaining CPE in the environment, effectively halting its spread to the upcoming batch.

#### 3.1.3 Duration of CPE outbreaks in flocks

**Fig. 4:**
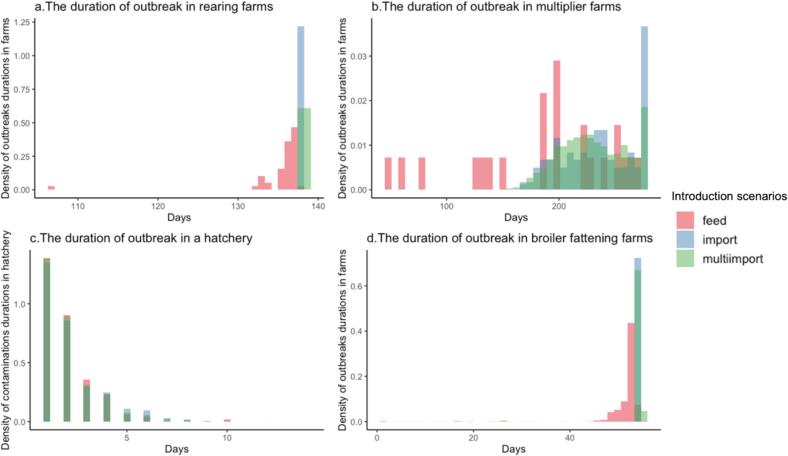
Distribution of the duration of CPE outbreaks in PS chickens, broiler chickens, and eggs contaminated with CPE. X-axis is the duration of outbreak in days and Y-axis is the number of durations counted from all 100 simulations. Blue- and green-colored bars show the number of colonized animals from single import and multiple import baseline models. Pink-colored bars show the number of colonized animals from the feed model. (For interpretation of the references to colour in this figure legend, the reader is referred to the web version of this article.)

The duration of CPE outbreaks in rearing farms and broiler farms is approximately equal to the length of one production round, regardless of the route of introduction (Fig. 4). For the import that is consistent with 1) the introduction by colonized animals at the start, either by live import (rearing farm) or colonized day-old chicks and 2) all-in-all out production system with 10 days downtime preventing spillover of colonization from one round to another. For feed, major outbreaks can only begin shortly after the start of a round due to the age-dependent susceptibility.

There is notable variation in the duration of colonization observed in multiplier farms compared to the length of their production round. In both single and multiple imports scenarios, the duration of colonization in multiplier farms ranges from 180 to 280 days (Fig. 4). This extended duration is due to the longer raising period in multiplier farms compared to rearing farms allows for variations in the timing when the flocks become completely rid of colonized PS chickens. However, we suspect that if the rearing farm extended the PS chicken raising period to be as long as that in the multiplier farm, the duration of colonization would be longer in rearing farms due to the higher susceptibility to colonization (and recolonization) in younger PS chickens compared to those in multiplier farms.

In the feed scenario, a small variation in the duration of CPE outbreaks in multiplier farms was observed, with a range of up to 50 days. This occurrence is attributed to a small number of colonized PS chickens from the rearing farm that was still colonized when they reached the multiplier farm. The relatively small population of colonized animals leads to a faster recovery in the farm, resulting in the complete elimination of contamination within a shorter time frame.

#### 3.1.4 CPE colonization in broiler flocks bound to slaughterhouse

Across all three scenarios, broiler farms experiencing a CPE outbreak will remain colonized until the time of slaughter (Fig. 4). However, the duration of the outbreak varies among sources due to differences in their introduction times. Outbreaks originating from feed have a slightly shorter duration because they are initiated during the middle of the production cycle, whereas those stemming from live imports begin at the beginning of the production cycle. With the reduction in colonization over time, the introduction from contaminated feed must have occurred before Day 8 to result in a major outbreak.

On the other hand, the number of colonized broiler chickens at slaughter is reduced to approximately 27 % of the peak number observed during the spread of the outbreak. This reduction in colonization is consistent across introduction routes. Among the scenarios, the feed introduction scenario shows the highest number of colonized broilers and broiler farms at the time of slaughter. This outcome is attributed to the fact that the feed introduction scenario results in a higher number of colonized flocks and broiler chickens compared to the other scenarios (refer to Sections 4.1.1 and 4.1.2 for more details).

**Fig. 5:**
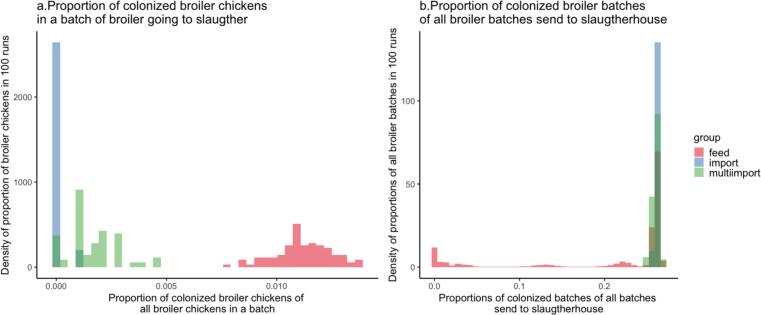
(a) The X-axis represents the proportion of broiler chickens colonized with CPE, relative to all broiler chickens in a batch. The Y-axis illustrates the frequency of these proportions across 100 simulations. (b) On the X-axis, the proportion of colonized batches from all broiler batches (6702 batches) sent to the slaughterhouse over a span of 9 years is displayed. The bars are colour-coded: blue and green represent proportions from the single import and multiple import models, while red indicates the proportion of colonized animals from the feed model. (For interpretation of the references to colour in this figure legend, the reader is referred to the web version of this article.)

Over the course of 9 years of simulations, a total of 6702 flocks of broiler chickens from 132 broiler farms were sent to slaughterhouses. Among these batches, the number of colonized batches was highest in feed introduction scenario ranging between 52 and 92 batches during 9 years. In the single import scenario, outbreaks in batches of broiler chickens are rare, resulting in 1 to 6 batches of broilers sent to slaughterhouses in 9 years (Fig. 5). However, when multiple introductions of CPE to a rearing farm occurred, the number of colonized batches sent to slaughterhouses increased significantly, ranging from 1 to 30 batches.

#### 3.1.5 Probability of detecting CPE outbreak with national surveillance

When considering the scenario of a single import into the rearing farm, only 10 % of the runs resulted in one or a series of outbreaks in broiler farms, impacting 1 to 6 farms (6 flocks). Consequently, the model predicts that in 90 % of the runs, the probability of detection will be 0, despite CPE having circulated in a rearing farm and multiplier farm. In these 1 to 6 colonized flocks, the probability that the colonized flock is being tested from national surveillance is 0.069. This probability represents the likelihood of the flock being selected for national sampling. Subsequently, the probability of detecting CPE within the colonized batch ($P_{\mathrm{posde}t_{i}})$ is approximately 0.92. As a result, the probability that CPE-colonized batch of broilers will be detected in the surveillance (${P_{\det}}_{i})$ is 0.064.

Consequently, the cumulative probability of detection over the studied period is 0 in 90 % of the runs and for the other 10 % varies between 0.022 and 0.064 when only one batch is contaminated in the entire run and 0.32 when 6 batches are contaminated. Thus, overall, the probability of detecting CPE after a single introduction with live birds is very low.

**Fig. 6:**
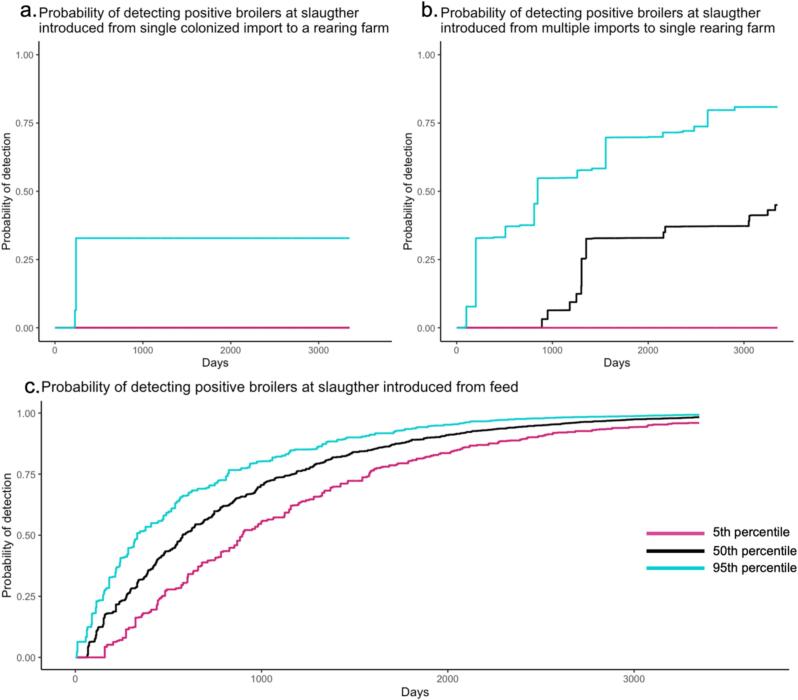
Cumulative probability of to detect CPE using Dutch National Surveillance Protocol. (a) displays the cumulative probability of detection introduced from a single batch of colonized PS chickens to a rearing farm. The (b) displays multiple batches of colonized import to a single rearing farm. The (c) displays the cumulative probability of detection introduced from contaminated feed. Black line represents the median, turquoise line represents the 5th percentile, and pink line indicates the 95th percentile probability of detection on a specific day. The right plot displays the cumulative probability of detection introduced from feed. (For interpretation of the references to colour in this figure legend, the reader is referred to the web version of this article.)

**Fig. 7:**
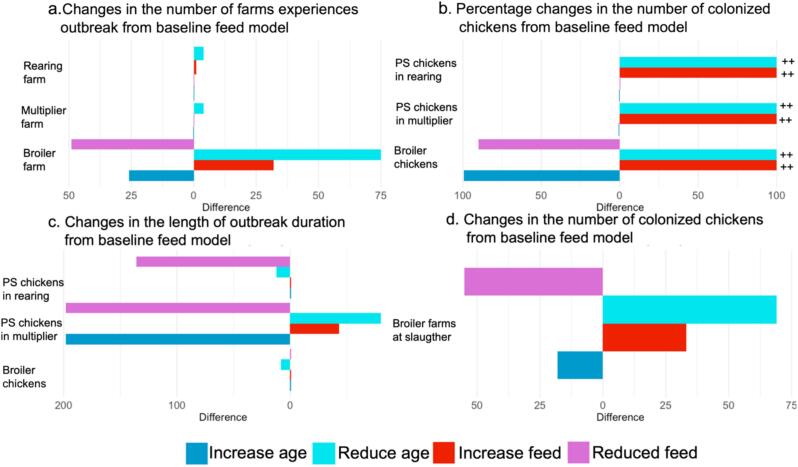
Sensitivity analysis results in feed scenario. Two parameters with significant correlation are included, the reduction in susceptibility to transmission due to age (ψ) and the rate of colonization from CPE contamination in feed on farms ($\omega_{\mathrm{ps}}\&\omega_{b}$). (a) tornado plot depicts the changes in number of colonized farms from baseline feed introduction model. (b) tornado plot shows the changes in number of colonized chickens in percentage from baseline feed introduction model. (c) tornado plot displays the days changes in the duration of outbreak in each farm type from baseline feed introduction model. (d) tornado plot shows the changes in the number of colonized broiler farms at slaughter time. The tornado plots display median number of mentioned outcome. The ++ sign in the percentage changes in the number of colonized chickens from baseline feed model means the changes is more than 100 %.

In the scenario with multiple imports into a rearing farm, the probability of detecting a CPE outbreak in a batch of broilers (${P_{\det}}_{i})$ is the same as in the single import scenario. Here, 91 of the 100 runs resulted in positive flocks of broilers, with the total number of positive farms varying between 8 and 24. Consequently, the cumulative probability of detection across the simulated period varied between runs (Fig. 6: left plot). Nevertheless, overall the probability of detection is higher than in the single import scenario, with a maximum cumulative probability of detection ranging from 0 to 0.81, with the 50 percentile at 0.45. Although the probability of detection is higher in this scenario than in the single import, also here there is a considerable probability of missing CPE in the surveillance and time to detect is generally long. (See Fig. 7.)

The continuous exposure of feed leads to different dynamics in the detection probability for import scenarios (Fig. 6). The series of outbreaks originating from feed are quite similar in the runs due to ongoing exposure to contaminated feed in all broiler farms. The cumulative probability of detection ranges from 0.95 to 0.99, with the 50 percentiles at 0.98. The time it takes to detect contaminated batches with a cumulative probability of 0.95 ranges from 1837 to 2935 days and a median of 2365 days after the first broiler chickens are contaminated.

### 3.2 Sensitivity analysis

In the feed model, we identified the strongest correlation between the age-associated reduction in transmission (ψ) to all output variables (Table 7; Supplementary material). Decreasing the reduction in susceptibility to transmission due to age (ψ) (resulting in higher probability of colonization at higher age) resulted in a significant increase in all output variables, as all animals were equally susceptible to CPE introduction. Doubling the reduction in susceptibility to transmission due to age (ψ) dramatically reduces all the output variables (Table 7).

**Table 7:** The results of a what-if analysis, including the number of broiler farms with major outbreaks at slaughter, the number of colonized broiler chickens at slaughter, the frequency of outbreaks in all broiler farms, and the day that the cumulative probability of detection will reach 0.95. * NA days means the cumulative probability of detection did not reach 0.95 within 9 years.

What-if analysis for single import model				
Scenarios	Number of farms with outbreak of CPE at slaughter	Number of broiler chickens colonized at slaughter	Frequency of outbreak in broiler farms	Days that the cumulative probability of detecting contaminated batch reached 0.95*
Baseline	0 [0;6]	0 [0, 101,814]	1 [1,1]	NA
All batches to rearing farms has contagious PS chickens	106.5 [96.9, 121]	5.21 E06 [4.57 E06, 6.64 E06]	2 [1, 5]	82 [19, 244]
Alternate batches to rearing farms has contagious PS chickens	70 [50.4, 94.95]	1.80 E06 [1.34 E06, 3 E06]	1 [1,3]	528 [213, 809]
Every year, a batch to broiler farms has contagious chickens	132 [132,132]	29.08 E06 [29.1 E 06, 29.2 E06]	8 [8, 8]	91 [89, 91]
Every batch to broiler farms has contagious chickens	132 [132, 132]	177.9 E06[ 177.6 E06, 178.4 E06]	49 [42, 53]	56 [56, 56]
Broilers are raised for 42 days	0 [0, 4.849]	0 [0, 113,080]	1 [1, 1]	NA
The environmental transmission rate is 10-fold due to antibiotics	0 [0, 6]	0 [0, 129,057]	1 [1, 1]	NA
What-if analysis for feed model				
	Number of farms with outbreak of CPE at slaughter	Number of broiler chickens colonized at slaughter	Frequency of outbreak in broiler farms	Days that the cumulative probability of detecting contaminated batch reached 0.95*
Baseline	56 [45, 64]	1.4 E06 [9.02 E05,1.43 E06]	1 [1,2]	754 [357,1063]
Broilers are raised for 42 days	53.5 [46.9, 61.85]	1.6 E06 [1.3 E06,1.8 E06]	1 [1,3]	671 [389, 734]
The environmental transmission rate is 10-fold due to antibiotics	58.5 [54, 63.55]	1.67 E06 [1.44 E06, 2 E06]	1 [1,2]	313 [577, 789]
The exposure to feed in broiler farms is limited to 13 farms (10 %)	4 [2.45, 8]	765 [392, 1871]	1 [1,3]	NA

Furthermore, the rate of colonization from CPE contamination in feed on farms ($\omega_{\mathrm{ps}}\&\omega_{b}$) demonstrated a notable correlation with the output related to broiler farms, although its impact was less pronounced than the reduction in transmission (ψ). When the rate of colonization from CPE contamination in feed on broiler farms ($\;\omega_{b}$) doubled, the number of broiler farms with outbreaks increased by 56 %. Conversely, a 90 % reduction in the rate of colonization from CPE contamination in feed on farms ($\;\omega_{b}$) eliminated the number of broiler farms experiencing outbreaks in other words, no outbreaks of CPE.

Across all outcomes, the colonization coefficient of CPE (β*)* showed a relatively minor influence on the results. Further sensitivity analysis showed that the other parameters rate of CPE excretion into the environment of the animal's pen (θ), rate of excreted CPE survival (ρ) and minimum susceptibility (z) – also had limited effects on the outcomes.

In the single import model, the recovery rate (γ) showed the strongest correlation with the number of broiler farms experiencing series of outbreaks compared to other parameters. Additionally, the reduction in transmission (ψ) also exhibits a moderate but notable correlation to the number of broiler farms experiencing series of outbreaks. Doubling both parameters did not result in an increased number of broiler farms with outbreaks. However, a tenfold reduction in the recovery rate (γ) and the reduction in transmission (ψ) led to a significant increase in the number of affected broiler farms, ranging from 89 to 118 farms (recovery rate) and 5 to 29 farms (reduction transmission), respectively. We see the same strong correlation of the recovery rate (γ) and the reduction in transmission (ψ) to the output of CPE-positive broilers at the slaughterhouse, the number of colonized broiler farms, and the prevalence of colonized broiler farms.

### 3.3 What-if analysis

#### 3.3.1 Import of colonized birds: Scenarios 1 to 4

The increased number of imports of contagious PS chickens to multiple rearing farms has a significant impact on the outbreak in broiler farms, resulting in a higher number of colonized broilers at slaughter (see Table 7). The more frequently contagious imports occur in rearing farms, the greater the number of broilers that become colonized.

The import of one-day-old broiler chickens from hatcheries outside the Netherlands would be a major risk of CPE introduction in broiler farms. Even a single import of contagious one-day-old broiler chickens per broiler farm per year has resulted in the colonization of all broiler farms, irrespective of the import frequency. This highlights the high susceptibility of broiler farms to a major outbreak from just one import event.

While the colonization of broiler farms remains constant regardless of the import frequency, the number of colonized broiler chickens at slaughter is directly proportional to the frequency of import. This means that the more frequent the contagious imports, the higher the number of broiler chickens that become colonized at the time of slaughter. To illustrate, both Scenario 3, with one imported batch of colonized broiler chickens per farm per year, and Scenario 4, with all imported broiler chicken batches being colonized per farm per year, result in the same total number of contagious broiler chickens imported from the hatchery per farm per year. Despite this consistency, the increased frequency of import in the fourth scenario leads to a higher overall number of colonized broiler chickens, reinforcing the correlation between import frequency and colonization rate.

Lastly, an increased number of imports raises the probability of detection based on national surveillance efforts due to the increased number of colonized chickens at slaughter. In the single import model, the probability of detection did not exceed 0.33. However, when the import of colonized PS chickens and broiler chickens increases, the probability of detection reached 0.95. The import of colonized broiler chickens to broiler farms significantly impacts the spread, leading to a probability of detection of 0.95 within 55 days after the broiler chickens enter the broiler farms. Other scenarios may take a longer time to achieve 0.95 probability of detection, but will not exceed 2 years.

#### 3.3.2 Reducing raising time in broiler farm and administering antibiotics: Scenarios 5 and 6

When broiler chickens are slaughtered at the age of 42 days instead of 56 days, both the single import model and the feed model show no significant changes in output (Table 7). However, the total number of colonized broiler chickens at slaughter was slightly higher when the raising time was reduced to 42 days, as the animals tend to lose their colonization as they age.

Similarly, in the 6th scenario, where the environmental transmission rate was increased by tenfold, simulating the effect of antibiotic treatment on transmission (β), the what-if analysis reveals slight increases in the number of colonized broiler chickens at slaughterhouses.

#### 3.3.3 Localizing the exposure to feed to 10 % of broiler farms

To test an alternative assumption in our analysis, we explored a scenario where we do not assume CPE homogeneously spreads to all feed mill in all farms. Instead, we examined the impact of localized introduction of CPE to a specific subset of feed mill in broiler farm. When the exposure to feed was restricted to 13 farms, several key outputs exhibited notable declines. The number of broiler farms experiencing major CPE outbreaks decreased from 46 to 2 farms, even though the number of colonized broiler farms varied, and in some runs, it closely resembled the results of the feed model in terms of major outbreaks. The number of colonized broiler chickens at slaughter saw a substantial reduction of 99.99 % due to the reduction in the number of colonized broiler farms at slaughter. This significant reduction in colonized broiler chickens effectively reduced the cumulative probability of detection, which ranged between 0.002 and 0.0147, and never reached a probability of 0.95 during the simulation. Consequently, this small probability of detection implies that broiler farms with contaminated feed, producing colonized broiler chickens, may remain undetected.

## 4 Discussion

The transmission simulation model presented in this study shows remarkable differences in dynamics between import of colonized animals and use of contaminated feed. Single imports are expected not to result in contaminated batches of broilers in most cases, despite circulation of CPE in rearing and multiplier PS birds. However, if contaminated broiler batches do arise, they have a low probability of being detected by the Dutch national AMR surveillance program. On the other hand, when multiple imports are involved, the probability of detection increases, and can reach a 0.8 (95th percentile) over a 10-year period. In the case of contaminated feed, the proportion of contaminated broilers is at its highest, as is the cumulative probability of detection reaches 0.99.

A crucial assumption in our model is the age-dependent susceptibility. Our sensitivity analysis has demonstrated that the model's outcomes are particularly sensitive to this parameter. Even slight reductions in this parameter can lead to significant changes in outcomes, ranging from nearly 0 to high values. This parameter limited the possibility of outbreaks to few days when the animals are of young ages. As animal matured, its susceptibility to CPE colonization drop to low levels. We see this phenomenon in real farms where matured animals have lower prevalence of resistance bacteria, part of the reason being the stable gut microbiome in broiler chickens [[32], [33], [34], [35], [36]].

When a farm is initially exposed to a small number of live animals through imports, CPE rapidly spreads to almost the entire flock (98–99 %) within three days, regardless of whether it is a rearing or broiler farm. On the contrary, the transmission through contaminated feed is less efficient in terms of spreading within a farm. In cases of major outbreaks in rearing and broiler farms, it takes 7–14 days to reach its peak, affecting 25 %–98 % of the animals in the farm. As animals age, their susceptibility to CPE colonization decreases. Therefore, the critical factor influencing the impact of the introduction lies in the timing of contagious chickens introduced into the batch. According to the model used here, colonization by contaminated feed resulting in a major outbreak can only take place in animals younger than 8 days. While the model assumed homogeneous mixing of all animals throughout the barn, the number of colonized live animals is likely to be reduced. However, the exposed barn will still experience a major outbreak and could make its way to the slaughterhouse.

In the import scenario, a single batch of 40 colonized PS chickens and broiler chickens are introduced to the farm at Day 1of a round, facilitating rapid spread throughout 99 % of the batch. Conversely, when colonization occurs through exposure to contaminated feed, a delay ensues, leading to a slower transmission rate as other broilers mature. For instance, if one PS chicken ingests CPE-contaminated feed and becomes colonized on Day 3, CPE can spread to approximately 72 % of the batch. However, if a PS chicken becomes colonized on Day 8 through feed exposure, it will not result in a major outbreak, affecting only 0.2 % of the batch.

The dynamics of CPE outbreaks in the broiler production system are influenced by various factors, including farm structure and biosecurity measures, leading to different outbreak characteristics. The broiler production system operates on an all-in-all-out basis, wherein all colonized animals must leave the farm before a new batch of animals arrives. As a result, outbreaks in rearing, multiplier, and broiler farms tend to die out between two production rounds. The downtime between batches effectively prevents continuous exposure to CPE contamination to the following batch of animal entering the same farm [37,38].

A single introduction into the rearing farm remains contained within that specific rearing farm and its corresponding multiplier farm because all animals are transported to the same multiplier farm. Although the same batch of PS chickens may supply eggs to a hatchery for a long period of up to 280 days, only a few contaminated eggs will be transported to the hatchery and even fewer broiler chickens will be colonized after hatching. Thus, outbreaks in broiler farms are rare and remain contained within one round. We refer to this outbreak characteristic as sporadic outbreaks.

Continuous or frequent exposure to CPE is crucial to achieve widespread and continuous CPE outbreaks in a significant number of farms. This assumes that transmission occurs primarily through direct animal contact with food and other animals. We refer to this continuing series of outbreaks as endemic outbreaks. Endemic outbreaks of CPE occur when farms are exposed continuously or frequently to CPE, especially during the early phase of the production round.

In a multiple imports model, compared to the single import model, the occurrence of outbreaks in all farm types is notably more frequent, which results in endemic outbreaks in broiler farms. Rearing farms transport to two multiplier farms in alternate rounds, leading to annual outbreaks in both connected multiplier farms. The doubled number of multiplier farms with repeated outbreaks results in a higher number of contaminated eggs in the hatchery. Consequently, the continuous influx of contaminated eggs from both multiplier farms, combined with the greater number of contaminated eggs, leads to all 100 runs experiencing outbreaks in at least one broiler farm.

Still, colonization due to multiple imports remained contained in less than 20 % of the total broiler farms (8 to 24 broiler farms) in the simulation. The reason is that horizontal between-farm spread does not occur in our model. Feed, on the other hand, resulted in colonization of 30 to 40 % of the broiler farms. This is due to the continuous exposure to feed in all farms.

Looking at the current information available from EU and national surveillance [7,8], a single contaminated import is a likely scenario. Detection of CPE in broiler production at slaughter has been rare in the EU national surveillance, with only 0.04 % or 3 positive samples out of 8530 [4]. This reflects our scenario for contamination in imported PS chickens, where we only see sporadic outbreaks in broilers with a very small probability of detection in the current surveillance program. Consequently, these few outbreaks might remain undetected in the national surveillance. The multiple import scenario is currently unlikely, as no CPE cases have been detected in Dutch livestock. However, it serves to predict what would happen if a source farm abroad were to become endemically colonized.

However, if the feed scenario as simulated here would be true, we would expect to detect outbreaks by the current Dutch surveillance. Although the presence of *E. coli* in feed has been demonstrated [39], no data regarding contamination with *Enterobacteriaceae* carrying CPE are available. CPE contamination in feed may occur through cross-contamination and from the environment [40,41]. The sensitivity analysis shows that the model is sensitive to the degree of feed contamination; reducing the exposure rate of feed in farms by 90 % reduces the number of broiler farms with CPE outbreaks at slaughter (Section 4.2) considerably.

The absence of detection in the current situation might have four possible explanations. Firstly, the concentration of CPE in the feed may be lower than assumed here. Secondly, the dose needed for colonization could be higher in real life. Both reasons will lead to a lower probability of birds being colonized. Thirdly, farms exposed to contaminated feed might be limited to only a few farms, such that detection is less probable because the flock is not sampled at slaughter. Lastly, there is no CPE contamination in feed at all. This last explanation reflects the outline of the Dutch broiler production system, which effectively limits the transmission of colonized chickens to a few farms.

A shift of target in sampling focus is advisable to enhance the probability of detecting potential outbreaks. Directing sampling efforts toward rearing farms is a prudent approach, given that these farms represent the point of introduction for CPE carried by live imports. This introduction, often resulting in sporadic outbreaks, carries a higher risk of escaping detection when it reaches broiler farms. Focusing on rearing farms, where live import birds initially enter the production cycle, enables prompt CPE identification before further spread. If available, surveillance should be specifically targeted at rearing farms that import PS chickens from outside the Netherlands. Additionally, rearing farms constitute a relatively smaller subset of the total farms within the industry compared to broiler farms, making comprehensive coverage achievable with fewer samples.

Sensitivity analysis has identified key parameters that greatly influence the persistence of CPE within the broiler population. An important variable with considerable uncertainty is the rate at which susceptibility reduces with age (ψ). The decline in susceptibility assumed here was linked to the decreasing trend of resistant extended-beta-lactamase-producing Enterobacteriaceae (ESBL) in broiler farms [35]. Nevertheless, as no dynamics of CPE have been observed within a broiler farm, it could be worthwhile to explore this for CPE. In the case of young broiler chickens, their gut is susceptible to colonization by various bacteria [42]. As they age, their gut exhibits a less diverse but stable and complex bacterial composition, enabling the animals to become more resilient against the invasion of exogenous bacteria [[42], [43], [44], [45]]. This protective effect of a stable gut microbiome against ESBL is observed in broiler chickens [35]. Furthermore, recent experiments involving CPE and ESBL transmission among one-day-old broiler chickens have shown a similar trend in bacterial composition, particularly as the chickens grow older. Both CPE and ESBL groups tend to converge toward similar bacterial complexity after 14 days in the experiment [20]. This convergence in gut composition could indicate the presence of a stable gut microbiome that acts preventively against the colonization of resistant bacteria.

The preventive effect of reduction in transmission rate may change in the presence of antibiotics. In Dankittipong et al. (2023) a specific antibiotic (amoxicillin) was administered to evaluate the impact of antibiotics on transmission. They found that broilers treated with amoxicillin exhibited a more diverse gut microbiome, indicating an opportunity for various bacteria species to grow after the disruption of the gut by the antibiotics [20]. Although the bacteria composition in both amoxicillin-treated and untreated groups eventually converged, we believe that stability was achieved primarily because amoxicillin was only present for a short time period and quickly degraded.

Studies by Rama et al. (2016) and Fairchild et al. (2005) also examined the effects of conventional antibiotics and tetracycline on antibiotic-resistant bacteria in 4-week-old broilers [46,47]. They found that older broilers consistently maintained antibiotic-resistant bacteria due to continuous disruption of the gut microbiome by antibiotics. Transitioning to a simulation, a what-if analysis scenario was introduced where the transmission rate was artificially increased tenfold, mimicking antibiotic use. The outcome demonstrated a 14 % increase in the number of broiler chickens at slaughter, although the number of colonized flocks at slaughter did not exhibit a significant rise. This outcome therefore suggested a restricted impact of antibiotics treatment to the acceleration of CPE spread. A reference to a One Health study [48] adds weight to the argument, suggesting that a 1 % increase in antibiotic usage corresponds to a modest 0.2 to 0.4 % escalation in resistance in animals. In conclusion, sensitivity analysis and what-if analysis reveal the complex interplay between age-related reduction in transmission rates, antibiotic treatments, and their collective impact. It asserts that the preventive efficacy of reducing transmission rates with age is not absolute and should be carefully evaluated in conjunction with antibiotic treatments.

This simulation study extends our comprehension of the emergence of resistant bacteria in broiler production and assesses the probability of detection. The simulation highlights the necessity of accurately estimating highly influential variables, such as the contamination and distribution of CPE in animal feed, as well as the reduction in the probability of colonization over time. The foremost action is monitoring the feed to establish whether CPE is present. After aligning important variables with the real situation, the model can be readily extended to investigate other surveillance programs, including sampling other types of poultry and varying sampling sizes and frequencies.

Recognizing that the transmission dynamics of CPE introduced through feed could be a major factor in its introduction and spread, it is imperative to gain a clear understanding of CPE contamination levels in feed and the precise CPE dosage required to initiate colonization. Routine collection of commensal bacteria in feed and CPE dose-response experiments will improve our transmission model accuracy in broiler production. For example, we currently lack knowledge about CPE's specific entry point into the feed mill system. Unlike imported livestock, where efforts can be focused on specific farm types that receive imports (rearing farms), once we ascertain the concentration and distribution of CPE in feed, we can pinpoint the timing and types of farms most likely to produce a higher number of colonized broilers. Additionally, feed serves as a source of CPE for other meat-producing animals like pigs, making it essential to obtain clear data on CPE concentration in feed [18]. This information helps assess the risks associated with antibiotic-resistant bacteria in the feed supply chain and develop targeted strategies for animal health and food safety.

By investigating two different routes of CPE introduction, import of live birds and contaminated feed, and assessing their impact on bacterial spread, this study is a steppingstone toward building an active surveillance strategy for the early detection of emerging CPE colonization in the broiler population. As a first step, the current Dutch surveillance program was assessed regarding its potential to detect CPE incursions at an early stage. Ultimately, this research will contribute to improved control and prevention measures in the broiler production system.

## CRediT authorship contribution statement

**N. Dankittipong:** Visualization, Validation, Methodology, Investigation, Formal analysis, Conceptualization, Writing – review & editing, Writing – original draft. **J.A. Stegeman:** Supervision, Project administration, Funding acquisition, Formal analysis, Conceptualization, Writing – review & editing. **C.J. de Vos:** Supervision, Conceptualization, Writing – review & editing. **J.A. Wagenaar:** Supervision, Conceptualization, Writing – review & editing. **E.A.J. Fischer:** Validation, Methodology, Formal analysis, Conceptualization, Writing – review & editing.

## Declaration of competing interest

None.

## Data Availability

Data will be made available on request.

## Appendix A Supplementary data

Supplementary material Image 1

## References

[1] Anderson R., Boerlin P. (2020). Carbapenemase-producing Enterobacteriaceae in animals and methodologies for their detection, the. Can. J. Vet. Res..

[2] Irrgang A., Tausch S.H., Pauly N., Grobbel M., Kaesbohrer A., Hammerl J.A. (2020). First detection of ges-5-producing escherichia coli from livestock—an increasing diversity of carbapenemases recognized from german pig production. Microorganisms.

[3] Levi G., Lurie-Weinberger M., Keren-Paz A., Andremont A.O., Schwartz D., Carmeli Y. (2022). Unraveling the diversity of co-colonization by CPE. Microorganisms.

[4] European Centre for Disease Prevention and Control (2014).

[5] Köck R., Daniels-Haardt I., Becker K., Mellmann A., Friedrich A.W., Mevius D., Schwarz S., Jurke A. (2018). Carbapenem-resistant Enterobacteriaceae in wildlife, food-producing, and companion animals: a systematic review. Clin. Microbiol. Infect..

[6] Mughini-Gras L., Dorado-García A., van Duijkeren E., van den Bunt G., Dierikx C.M., Bonten M.J.M., Bootsma M.C.J., Schmitt H., Hald T., Evers E.G., de Koeijer A., van Pelt W., Franz E., Mevius D.J., Heederik D.J.J. (2019). Attributable sources of community-acquired carriage of Escherichia coli containing β-lactam antibiotic resistance genes: a population-based modelling study. Lancet Planet Health.

[7] MARAN (2021).

[8] Nielsen S.S., Bicout D.J., Calistri P., Canali E., Drewe J.A., Garin-Bastuji B., Gonzales Rojas J.L., Gortázar C., Herskin M., Michel V., Miranda Chueca M.Á., Padalino B., Pasquali P., Roberts H.C., Spoolder H., Ståhl K., Velarde A., Viltrop A., Winckler C., Baldinelli F., Broglia A., Kohnle L., Alvarez J. (2022). Assessment of listing and categorisation of animal diseases within the framework of the animal health law (Regulation (EU) No 2016/429): antimicrobial-resistant *Escherichia coli* in dogs and cats, horses, swine, poultry, cattle, sheep and goats. EFSA J..

[9] ECDC (2018). The European Union summary report on antimicrobial resistance in zoonotic and indicator bacteria from humans, animals and food in 2016. EFSA J..

[10] MARAN (2020).

[11] Leverstein-van Hall M.A., Dierikx C.M., Cohen Stuart J., Voets G.M., van den Munckhof M.P., van Essen-Zandbergen A., Platteel T., Fluit A.C., van de Sande-Bruinsma N., Scharinga J., Bonten M.J.M., Mevius D.J. (2011). Dutch patients, retail chicken meat and poultry share the same ESBL genes, plasmids and strains. Clin. Microbiol. Infect..

[12] Furusawa M., Widgren S., Evers E.G., Fischer E.A.J. (2024). Quantifying health risks from ESBL-producing Escherichia coli in Dutch broiler production chains and potential interventions using compartmental models. Prev. Vet. Med..

[13] Salines M., Andraud M., Rose N., Widgren S. (2020). A between-herd data-driven stochastic model to explore the spatio-temporal spread of hepatitis e virus in the French pig production network. PLoS One.

[14] Sørensen A.I.V., Toft N., Boklund A., Espinosa-Gongora C., Græsbøll K., Larsen J., Halasa T. (2017). A mechanistic model for spread of livestock-associated methicillin-resistant Staphylococcus aureus (LA-MRSA) within a pig herd. PLoS One.

[15] Tuominen K.S., Sternberg Lewerin S., Widgren S., Rosendal T. (2023). Assessment of control measures against livestock-associated methicillin-resistant Staphylococcus aureus in a farrow-to-finish pig herd using infectious disease modelling. Animal.

[16] Sykes A.L., Galvis J.A., O’Hara K.C., Corzo C., Machado G. (2023). Estimating the effectiveness of control actions on African swine fever transmission in commercial swine populations in the United States. Prev. Vet. Med..

[17] Rosendal T., Widgren S., Ståhl K., Frössling J. (2020). Modelling spread and surveillance of Mycobacterium avium subsp. paratuberculosis in the Swedish cattle trade network. Prev. Vet. Med..

[18] Dankittipong N., Fischer E.A.J., Swanenburg M., Wagenaar J.A., Stegeman A.J., de Vos C.J. (2022). Quantitative risk assessment for the introduction of Carbapenem-resistant Enterobacteriaceae (CPE) into Dutch livestock farms. Antibiotics.

[19] Statistik, Statline, 2024 (n.d.).

[20] Dankittipong N., Alderliesten J.B., Van den Broek J., Dame-Korevaar M.A., Brouwer M.S.M., Velkers F.C., Bossers A., de Vos C.J., Wagenaar J.A., Stegeman J.A., Fischer E.A.J. (2023). Comparing the transmission of carbapenemase-producing and extended-spectrum beta-lactamase-producing Escherichia coli between broiler chickens. Prev. Vet. Med..

[21] Dame-Korevaar A., Fischer E.A.J., Stegeman A., Mevius D., van Essen-Zandbergen A., Velkers F., van der Goot J. (2017). Dynamics of CMY-2 producing E. Coli in a broiler parent flock. Vet. Microbiol..

[22] Huijbers P.M.C., Graat E.A.M., van Hoek A.H.A.M., Veenman C., de Jong M.C.M., van Duijkeren E. (2016). Transmission dynamics of extended-spectrum β-lactamase and AmpC β-lactamase-producing Escherichia coli in a broiler flock without antibiotic use. Prev. Vet. Med..

[23] Dierikx C.M., Van Der Goot J.A., Smith H.E., Kant A., Mevius D.J. (2013). Presence of ESBL/AmpC -producing Escherichia coli in the broiler production pyramid: a descriptive study. PLoS One.

[24] Schreuder J., Velkers F.C., Bouwstra R.J., Beerens N., Stegeman J.A., de Boer W.F., van Hooft P., Elbers A.R.W., Bossers A., Jurburg S.D. (2020). An observational field study of the cloacal microbiota in adult laying hens with and without access to an outdoor range. Anim. Microbiome.

[25] Projahn M., Daehre K., Roesler U., Friese A. (2017). Extended-spectrum-beta-lactamaseand plasmid-encoded cephamycinaseproducing enterobacteria in the broiler hatchery as a potential mode of pseudovertical transmission. Appl. Environ. Microbiol..

[26] Archer G., Cartwright L. (2017). Incubating and Hatching Eggs.

[27] Dankittipong N., Van den Broek J., de Vos C.J., Wagenaar J.A., Stegeman J.A., Fischer E.A.J. (2024). Transmission rates of veterinary and clinically important antibiotic resistant Escherichia coli: a meta- ANALYSIS. Prev. Vet. Med..

[28] Mostert P.F., Bos A.P., Van Harn J., Van Horne P., De Jong I.C. (2022). Environmental Impacts of Broiler Production Systems in the Netherlands. http://www.wageningenUR.nl/livestockresearch.

[29] De Wit G., Svet L., Lories B., Steenackers H.P. (2022).

[30] (2018). The European Union summary report on antimicrobial resistance in zoonotic and indicator bacteria from humans, animals and food in 2016. EFSA J..

[31] Kirkeby C., Halasa T., Gussmann M., Toft N., Græsbøll K. (2017). Methods for estimating disease transmission rates: evaluating the precision of Poisson regression and two novel methods. Sci. Rep..

[32] Jurburg S.D., Brouwer M.S.M., Ceccarelli D., van der Goot J., Jansman A.J.M., Bossers A. (2019). Patterns of community assembly in the developing chicken microbiome reveal rapid primary succession. Microbiologyopen.

[33] Kers J.G., Velkers F.C., Fischer E.A.J., Arjan Stegeman J., Smidt H., Hermes G.D.A. (2022). Conserved developmental trajectories of the cecal microbiota of broiler chickens in a field study. FEMS Microbiol. Ecol..

[34] Callaway T., Carr M., Edrington T., Anderson R., Nisbet D. (2009). Diet, *Escherichia coli* O157:H7, and Cattle: A Review after 10 Years. http://www.cimb.org.

[35] Dame-Korevaar A., Fischer E.A.J., van der Goot J., Velkers F., Ceccarelli D., Mevius D., Stegeman A. (2020). Early life supply of competitive exclusion products reduces colonization of extended spectrum beta-lactamase-producing Escherichia coli in broilers. Poult. Sci..

[36] Laube H., Friese A., von Salviati C., Guerra B., Rösler U. (2014). Transmission of ESBL/AmpC-producing Escherichia coli from broiler chicken farms to surrounding areas. Vet. Microbiol..

[37] K. De Reu, K. LuycKx, ELs Van CoiLLie, M. HeynDRicKx, J. DewuLf, SHaRon Maes, H. MaeRtens, Implications, Efficiency and Evaluation of Cleaning and Disinfection in Commercial Broiler farms, 2019. www.belplume.be.

[38] Luyckx K., Dewulf J., Van Weyenberg S., Herman L., Zoons J., Vervaet E., Heyndrickx M., De Reu K. (2014). Comparison of sampling procedures and microbiological and non-microbiological parameters to evaluate cleaning and disinfection in broiler houses. Poult. Sci..

[39] GMP+ (2020).

[40] Filippitzi M.E., Sarrazin S., Imberechts H., Smet A., Dewulf J. (2016). Risk of cross-contamination due to the use of antimicrobial medicated feed throughout the trail of feed from the feed mill to the farm. Food Addit. Contam. Part A Chem. Anal. Control Expo. Risk Assess..

[41] Barbosa Da Silva A., Back M., Daguer H., Palmeira M., Ploêncio L. Antunes De Sá, Molognoni L., Peripolli V., Bianchi I. (2019). Carry-over and contamination of veterinary drugs in feed production lines for poultry and pigs. Food Addit. Contam. Part A Chem. Anal. Control Expo Risk Assess..

[42] Ballou A.L., Ali R.A., Mendoza M.A., Ellis J.C., Hassan H.M., Croom W.J., Koci M.D. (2016). Development of the chick microbiome: how early exposure influences future microbial diversity. Front. Vet. Sci..

[43] Awad W.A., Mann E., Dzieciol M., Hess C., Schmitz-Esser S., Wagner M., Hess M. (2016). Age-related differences in the luminal and mucosa-associated gut microbiome of broiler chickens and shifts associated with campylobacter jejuni infection. Front. Cell. Infect. Microbiol..

[44] Rochegüe T., Haenni M., Mondot S., Astruc C., Cazeau G., Ferry T., Madec J.Y., Lupo A. (2021). Impact of antibiotic therapies on resistance genes dynamic and composition of the animal gut microbiota. Animals.

[45] Kim S., Covington A., Pamer E.G. (2017). The intestinal microbiota: antibiotics, colonization resistance, and enteric pathogens. Immunol. Rev..

[46] Novoa Rama E., Bailey M., Kumar S., Leone C., den Bakker H.C., Thippareddi H., Singh M. (2022). Prevalence and antimicrobial resistance of Salmonella in conventional and no antibiotics ever broiler farms in the United States. Food Control.

[47] Fairchild A.S., Smith J.L., Idris U., Lu J., Sanchez S., Purvis L.B., Hofacre C., Lee M.D. (2005). Effects of orally administered tetracycline on the intestinal community structure of chickens and on tet determinant carriage by commensal bacteria and campylobacter jejuni. Appl. Environ. Microbiol..

[48] Rahman S., Hollis A. (2023). The effect of antibiotic usage on resistance in humans and food-producing animals: a longitudinal, one health analysis using European data. Front. Public Health.

