# Supplementary material for: Investigating a propagation of emerging carbapenemase-producing Enterobacteriaceae in Dutch broiler production pyramid through stochastic simulation
Source: One Health. 2024 Nov 26;19:100945. doi: 10.1016/j.onehlt.2024.100945 (PMC11635706; doi:10.1016/j.onehlt.2024.100945)
Supplement: Supplementary file 1 — Supplementary material [file mmc1.docx]

**Supplementary materials**

**Appendix 1: Decay rate from literatures.**

Table S1. Decay of Escherichia coli outside a live host. Abbreviations: fc: field capacity.

| ID | Temperature (°C) | Humidity | pH | Environment | Decay rate (per day) | Reference |
| --- | --- | --- | --- | --- | --- | --- |
| 1 | Autumn |  |  | Lab | 0.102 | (Burrows and Rankin, 1970) |
| 2 | Autumn |  |  | Lab | 0.287 | (Burrows and Rankin, 1970) |
| 3 | 4 |  | 7 | Lab | 0.686 | (Kovács and Tamási, 1977) |
| 4 | January |  |  | Lab | 0.109 | (Rankin and Taylor, 1969) |
| 5 | 26 |  | 7.4 | Soil | 0.896 | (Klein and Casida, 1967) |
| 6 | 10 |  | 7.4 | Soil | 0.195 | (Klein and Casida, 1967) |
| 7 |  |  |  | Soil | 0.115 | (Mallmann and Litsky, 1951) |
| 8 |  |  | 7 | Soil | 0.371 | (Van Donsel et al., 1967) |
| 9 |  |  |  | Soil | 0.143 | (Mallmann and Litsky, 1951) |
| 10 |  | 1/3 bar | 6.16 | Soil | 0.473 | (Tate, 1978) |
| 11 |  | saturated | 6.64 | Soil | 0.839 | (Tate, 1978) |
| 12 |  | 100% fc | 6.16 | Soil | 0.796 | (Tate, 1978) |
| 13 |  | flooded |  | Soil | 0.382 | (Tate, 1978) |
| 14 | 0 |  |  | Inoculated water | 0.192 | (Mitchell, 1968) |
| 15 | 10 | 60% fc |  | Swine manure-amended soil | 0.22 | (Rogers et al., 2011) |
| 16 | 10 | 80% fc |  | Swine manure-amended soil | 0.19 | (Rogers et al., 2011) |
| 17 | 25 | 60% fc |  | Swine manure-amended soil | 0.40 | (Rogers et al., 2011) |
| 18 | 25 | 80% fc |  | Swine manure-amended soil | 0.28 | (Rogers et al., 2011) |
| 19 | 10 | 60% fc |  | Beef manure-amended soil | 0.17 | (Rogers et al., 2011) |
| 20 | 10 | 80% fc |  | Beef manure-amended soil | 0.15 | (Rogers et al., 2011) |
| 21 | 25 | 60% fc |  | Beef manure-amended soil | 0.33 | (Rogers et al., 2011) |
| 22 | 25 | 80% fc |  | Beef manure-amended soil | 0.37 | (Rogers et al., 2011) |
| 23 | Optimal condition to rear broilers | | | Broiler pen floor | 0.0 | (van Bunnik et al., 2014) |

**Appendix 2: queries to get demographic data from CBS**

**Supplementary XII : Queries to retrieve import livestock of interest from cbs.nl**

URL: <https://opendata.cbs.nl/statline/portal.html>

- Click “Kies thema” at the top of the page
- Click the following options internationale handel> handel; goederen> goederensoorten, landen per jaar> natuur, voeding en tabak; jaar
- Click "Preview data" then Select the following animal species from the drop down “goederensoorten natuur, voeding en tabak”

**0102291000**: Cattle, live, with a weight of <= 80 kg (excl. pure-bred breeding cattle)

**0102900500**: Cattle/ domestic animals/live weighing <= 80 kg (excl. pure-bred breeding animals)

**0103100000**: Pure-bred breeding pigs

**0103911000**: Pigs/ domestic animals/ live pigs weighing <50 kg (excl. pure-bred breeding animals

**0103921100**: Sows /domestic animals /live ..."who have farrowed at least once, weighing> = 160 kg (excl. pure-bred breeding animals)

**0105111900**: Female breeding chicks of chickens/ poultry/weighing <= 185 g (excl. those of laying breeds)

**0105119900**: Roosters and chickens/ poultry/ weighing <= 185 g (excl. those of laying breeds and excl. female and breeding chicks)

- Specify imported animals by select drop down “Onderwerp” > Invoerhoeveelheid
- Specify countries of import (European Member states in our analysis) by select drop down “Landen”

Supplementary material 3: Number of farms with major outbreaks,

**Appendix 3. Sensitivity analysis**

Table S2. Output for the sensitivity analysis in one import baseline

|  |  | Number of farms with outbreak of CPE | | Number of chickens colonized with CPE | | Duration of outbreak | | Frequency of outbreak per farm | |
| --- | --- | --- | --- | --- | --- | --- | --- | --- | --- |
| Parameter | Farm | n.farm | difffarm | n.animal | difffarm | Days | difffarm | Times | difffarm |
| gamma_d | rearing | 1[1,1] | 0[0,0] | 39862.5[39846.9,39879.5] | -69.5[-72.0999999999985,-65.5500000000029] | 138[138,138] | 0[0,0] | 1[1,1] | 0[0,0] |
| gamma_0 | rearing | 1[1,1] | 0[0,0] | 40000[40000,40000] | 68[81,54.9499999999971] | 138[138,138] | 0[0,0] | 1[1,1] | 0[0,0] |
| agedecay_d | rearing | 1[1,1] | 0[0,0] | 35799.5[34608.65,36186.7] | -4132.5[-5310.35,-3758.35000000001] | 138[138,138] | 0[0,0] | 1[1,1] | 0[0,0] |
| agedecay_0 | rearing | 1[1,1] | 0[0,0] | 40239.5[40217.4,40263.8] | 307.5[298.400000000001,318.75] | 138[138,138] | 0[0,0] | 1[1,1] | 0[0,0] |
| min_d | rearing | 1[1,1] | 0[0,0] | 39933[39912.25,39943.85] | 1[-6.75,-1.19999999999709] | 138[138,138] | 0[0,0] | 1[1,1] | 0[0,0] |
| min_0 | rearing | 1[1,1] | 0[0,0] | 39932.5[39921.25,39947.65] | 0.5[2.25,2.59999999999854] | 138[138,138] | 0[0,0] | 1[1,1] | 0[0,0] |
| theta_d | rearing | 1[1,1] | 0[0,0] | 39929.5[39920.45,39941.75] | -2.5[1.44999999999709,-3.30000000000291] | 138[138,138] | 0[0,0] | 1[1,1] | 0[0,0] |
| theta_0 | rearing | 1[1,1] | 0[0,0] | 39933[39912.25,39943.85] | 1[-6.75,-1.19999999999709] | 138[138,138] | 0[0,0] | 1[1,1] | 0[0,0] |
| rho_d | rearing | 1[1,1] | 0[0,0] | 39968.5[39961.45,39976.2] | 36.5[42.4499999999971,31.1499999999942] | 138[138,138] | 0[0,0] | 1[1,1] | 0[0,0] |
| rho_0 | rearing | 0[0,0] | -1[-1,-1] | 36.5[30.45,43.1] | -39895.5[-39888.55,-39901.95] | 138[116,138] | 0[-22,0] | 1[1,1] | 0[0,0] |
| beta_d | rearing | 1[1,1] | 0[0,0] | 39987[39982,39992.55] | 55[63,47.5] | 138[138,138] | 0[0,0] | 1[1,1] | 0[0,0] |
| beta_0 | rearing | 0[0,0] | -1[-1,-1] | 42[36,50.55] | -39890[-39883,-39894.5] | 134.5[97.6,138] | -3.5[-40.4,0] | 1[1,1] | 0[0,0] |
| gamma_d | multiplier | 0[0,0] | -1[-1,-1] | 14[11,18.1] | -779[-742.95,-817.9] | 49.5[30.45,77.05] | -186.5[-154.45,-200.95] | 1[1,1] | 0[0,0] |
| gamma_0 | multiplier | 1[1,1] | 0[0,0] | 40000[40000,40000] | 39207[39246.05,39164] | 278[278,278] | 42[93.1,0] | 1[1,1] | 0[0,0] |
| agedecay_d | multiplier | 1[1,1] | 0[0,0] | 625.5[585.95,653.8] | -167.5[-168,-182.2] | 236[193.1,278] | 0[8.200002,0] | 1[1,1] | 0[0,0] |
| agedecay_0 | multiplier | 1[1,1] | 0[0,0] | 40471[40446.85,40515.55] | 39678[39692.9,39679.55] | 278[278,278] | 42[93.1,0] | 1[1,1] | 0[0,0] |
| min_d | multiplier | 1[1,1] | 0[0,0] | 788.5[766.25,817.65] | -4.5[12.3,-18.35] | 234[181.55,274.85] | -2[-3.349999,-3.15000000000003] | 1[1,1] | 0[0,0] |
| min_0 | multiplier | 1[1,1] | 0[0,0] | 782[764.25,825.15] | -11[10.3,-10.85] | 229.5[187.25,278] | -6.5[2.3499,0] | 1[1,1] | 0[0,0] |
| theta_d | multiplier | 1[1,1] | 0[0,0] | 790[725.55,821.55] | -3[-28.4000000000001,-14.45] | 238.5[189.15,278] | 2.5[4.25,0] | 1[1,1] | 0[0,0] |
| theta_0 | multiplier | 1[1,1] | 0[0,0] | 788.5[766.25,817.65] | -4.5[12.3,-18.35] | 234[181,274.85] | -2[-3.349999,-3.150000] | 1[1,1] | 0[0,0] |
| rho_d | multiplier | 1[1,1] | 0[0,0] | 826[769.15,854.65] | 33[15.1999999999999,18.6500000000001] | 238.5[216.45,273.5] | 2.5[31.55,-4.5] | 1[1,1] | 0[0,0] |
| rho_0 | multiplier | 0[0,0] | -1[-1,-1] | 1[0,2.55] | -792[-753.95,-833.45] | 44[6.2,81.6] | -192[-178.7,-196.4] | 1[1,1] | 0[0,0] |
| beta_d | multiplier | 1[1,1] | 0[0,0] | 842.5[823.85,885.6] | 49.5[69.9,49.5999999999999] | 214.5[193,272.15] | -21.5[8.09999999999999,-5.85000000000002] | 1[1,1] | 0[0,0] |
| beta_0 | multiplier | 0[0,0] | -1[-1,-1] | 0.5[0,2.1] | -792.5[-753.95,-833.9] | 23[6,50] | -213[-178.9,-228] | 1[1,1] | 0[0,0] |
| gamma_d | hatchery | 0[0,0] | 0[0,0] | 0[0,0] | -10[-6,-15] | 0[0,0] | -2[-1,-5] | 0[0,0] | -11[-11,-11] |
| gamma_0 | hatchery | 1[1,1] | 1[1,1] | 3511[3430.05,3564.1] | 3501[3424.05,3549.1] | 283[280,285] | 281[279,280] | 285[285,285] | 274[274,274] |
| agedecay_d | hatchery | 1[1,1] | 1[1,1] | 9.5[5.8,14.65] | -0.5[-0.2,-0.350000000000003] | 1[1,2.85] | -1[0,-2.15] | 13[13,13] | 2[2,2] |
| agedecay_0 | hatchery | 1[1,1] | 1[1,1] | 3484[3423.15,3581.6] | 3474[3417.15,3566.6] | 281.5[281,284] | 279.5[280,279] | 286[286,286] | 275[275,275] |
| min_d | hatchery | 1[1,1] | 1[1,1] | 12[8.35,16.1] | 2[2.35,1.1] | 2[1,3] | 0[0,-2] | 13[13,13] | 2[2,2] |
| min_0 | hatchery | 1[1,1] | 1[1,1] | 9.5[5,12] | -0.5[-1,-3] | 1[1,3.1] | -1[0,-1.9] | 15[15,15] | 4[4,4] |
| theta_d | hatchery | 1[1,1] | 1[1,1] | 11[6.8,13] | 1[0.8,-2] | 1[1,4.75] | -1[0,-0.25] | 25[25,25] | 14[14,14] |
| theta_0 | hatchery | 1[1,1] | 1[1,1] | 12[8.35,16.1] | 2[2.35,1.1] | 2[1,3] | 0[0,-2] | 13[13,13] | 2[2,2] |
| rho_d | hatchery | 1[1,1] | 1[1,1] | 10[5.45,15.65] | 0[-0.550000000000001, 0.649999999999997] | 1[1,4] | -1[0,-1] | 15[15,15] | 4[4,4] |
| rho_0 | hatchery | 0[0,0] | 0[0,0] | 0[0,0] | -10[-6,-15] | 0[0,0] | -2[-1,-5] | 0[0,0] | -11[-11,-11] |
| beta_d | hatchery | 1[1,1] | 1[1,1] | 12.5[6.9,19.55] | 2.5[0.9,4.55] | 1[1,5.3] | -1[0,0.299999] | 27[27,27] | 16[16,16] |
| beta_0 | hatchery | 0[0,0] | 0[0,0] | 0[0,0] | -10[-6,-15] | 0[0,0] | -2[-1,-5] | 0[0,0] | -11[-11,-11] |
| gamma_d | broiler | 0[0,0] | 0[0,-6] | 0[0,0] | 0[0,-390269.95] | 0[0,0] | -54[-54,-54] | 0[0,0] | -1[-1,-1] |
| gamma_0 | broiler | 112[101.9,119.55] | 112[101.9,113.55] | 13163438.5[12275957,14732323] | 13163438.5[12275957.1, 14342053.15] | 54[54,55] | 0[0,1] | 2[1,3] | 1[0,2] |
| agedecay_d | broiler | 0[0,3.2999] | 0[0,-2.70000] | 0[0,132811.8] | 0[0,-257458.15] | 0[0,0] | -54[-54,-54] | 1[1,1] | 0[0,0] |
| agedecay_0 | broiler | 105.5[92.5,124.4] | 105.5[92.5,118.4] | 13031605[10270825,16141983] | 13031605[10270825.45, 15751713.1] | 54[54,55] | 0[0,1] | 1[1,3] | 0[0,2] |
| min_d | broiler | 0[0,3.74999] | 0[0,-2.25000] | 0[0,304316.7] | 0[0,-85953.2500000003] | 54[54,54] | 0[0,0] | 1[1,1] | 0[0,0] |
| min_0 | broiler | 0[0,0] | 0[0,-6] | 0[0,0] | 0[0,-390269.95] | 0[0,0] | -54[-54,-54] | 0[0,0] | -1[-1,-1] |
| theta_d | broiler | 0[0,6] | 0[0,0] | 0[0,507826.35] | 0[0,117556.4] | 54[54,54] | 0[0,0] | 1[1,1] | 0[0,0] |
| theta_0 | broiler | 0[0,3.7499] | 0[0,-2.2500000] | 0[0,304316.7] | 0[0,-85953.2500000003] | 54[54,54] | 0[0,0] | 1[1,1] | 0[0,0] |
| rho_d | broiler | 0[0,6] | 0[0,0] | 0[0,390928.9] | 0[0,658.95000000007] | 54[54,54] | 0[0,0] | 1[1,1] | 0[0,0] |
| rho_0 | broiler | 0[0,0] | 0[0,-6] | 0[0,0] | 0[0,-390269.95] | 0[0,0] | -54[-54,-54] | 0[0,0] | -1[-1,-1] |
| beta_d | broiler | 0[0,6] | 0[0,0] | 0[0,440411.85] | 0[0,50141.9] | 54[54,54] | 0[0,0] | 1[1,1] | 0[0,0] |
| beta_0 | broiler | 0[0,0] | 0[0,-6] | 0[0,0] | 0[0,-390269.95] | 0[0,0] | -54[-54,-54] | 0[0,0] | -1[-1,-1] |

Table S3. Output for sensitivity analysis from continuous exposure to feed.

|  |  | Number of farms with outbreak of CPE | | Number of chickens colonized with CPE | | Duration of outbreak | | Frequecy of outbreak per farm | |
| --- | --- | --- | --- | --- | --- | --- | --- | --- | --- |
| Farm | para | n.farm | difffarm | n.animal | difffarm | Days | difffarm | Times | difffarm |
| rearing | gamma_d | 0[0,1] | 0[0,-1] | 0[0,33918.15] | 0[0,-30052.65] | 136.5[136.05,136.95] | 0.5[48.85,-0.049] | 1[1,1] | 0[0,-1] |
| rearing | gamma_0 | 1[0,1.55] | 1[0,-0.450] | 15047.5[0,40038.55] | 15047.5[0,-23932.25] | 136.5[131.05,137] | 0.5[43.85,0] | 1[1,1] | 0[0,-1] |
| rearing | agedecay_d | 0[0,1] | 0[0,-1] | 0[0,128.15] | 0[0,-63842.65] | 137[137,137] | 1[49.8,0] | 1[1,1] | 0[0,-1] |
| rearing | agedecay_0 | 14[11.9,15] | 14[11.9,13] | 1123005[740053.7,1511038.6] | 1123005[740053.7,1447067.8] | 73[13,131] | -63[-74.2,-6] | 1[1,2] | 0[0,0] |
| rearing | min_d | 0[0,1.55] | 0[0,-0.450] | 0[0,27260.05] | 0[0,-36710.7500000001] | 135.5[135,136.85] | -0.5[47.8,-0.15000] | 1[1,2] | 0[0,0] |
| rearing | min_0 | 0[0,1.55] | 0[0,-0.450] | 0[0,53174.7] | 0[0,-10796.1] | 136.5[50.15,137] | 0.5[-37.05,0] | 1[1,1] | 0[0,-1] |
| rearing | omega_ps_d | 0.5[0,2] | 0.5[0,0] | 1450[0,74241.6] | 1450[0,10270.8] | 137[134.35,137] | 1[47.15,0] | 1[1,1] | 0[0,-1] |
| rearing | omega_ps_0 | 0[0,0] | 0[0,-2] | 0[0,0] | 0[0,-63970.8] | NA | NA | NA | NA |
| rearing | omega_b_d | 0.5[0,1] | 0.5[0,-1] | 2244.5[0,39257.2] | 2244.5[0,-24713.6] | 136[96.75,137] | 0[9.55,0] | 1[1,1] | 0[0,-1] |
| rearing | omega_b_0 | 0[0,1] | 0[0,-1] | 0[0,18463.85] | 0[0,-45506.95] | 134.5[134,135.85] | -1.5[46.8,-1.1500] | 1[1,1] | 0[0,-1] |
| rearing | theta_d | 0[0,1] | 0[0,-1] | 0[0,39652.9] | 0[0,-24317.9] | 135.5[132.45,136.85] | -0.5[45.25,-0.1500] | 1[1,1] | 0[0,-1] |
| rearing | theta_0 | 0[0,1.55] | 0[0,-0.450] | 0[0,27260.05] | 0[0,-36710.7500000001] | 135.5[135,136.85] | -0.5[47.8,-0.150] | 1[1,2] | 0[0,0] |
| rearing | rho_d | 0[0,1] | 0[0,-1] | 0[0,39652.9] | 0[0,-24317.9] | 135.5[132.45,136.85] | -0.5[45.25,-0.150000] | 1[1,1] | 0[0,-1] |
| rearing | rho_0 | 0[0,0] | 0[0,-2] | 0[0,1] | 0[0,-63969.8] | 12[5.7,12] | -124[-81.5,-125] | 1[1,1] | 0[0,-1] |
| rearing | beta_d | 0[0,1.55] | 0[0,-0.450] | 0[0,61727.05] | 0[0,-2243.75000000004] | 135.5[133.3,136.85] | -0.5[46.1,-0.15000] | 1[1,1] | 0[0,-1] |
| rearing | beta_0 | 0[0,0] | 0[0,-2] | 0[0,1] | 0[0,-63969.8] | 8.5[1.75,46.7] | -127.5[-85.45,-90.3] | 1[1,1] | 0[0,-1] |
| multiplier | gamma_d | 0[0,0] | 0[0,-2] | 0[0,21] | 0[0,-1350.8] | 57[57,57] | -141[-6.6,-204.4] | 1[1,1] | 0[0,0] |
| multiplier | gamma_0 | 1[0,1.55] | 1[0,-0.450] | 15047.5[0,40038.55] | 15047.5[0,38666.75] | 278[278,278] | 80[214.4,16.6] | 1[1,1] | 0[0,0] |
| multiplier | agedecay_d | 0[0,0] | 0[0,-2] | 0[0,3] | 0[0,-1368.8] | NA | NA | 1[1,1] | 0[0,0] |
| multiplier | agedecay_0 | 29[26.45,30] | 29[26.45,28] | 2,587,216.5[2190395.05,3090142.55] | 2587216.5[2190395.05,3088770.75] | 208[11.4,279] | 10[-52.2,17.6] | 1[1,3] | 0[0,2] |
| multiplier | min_d | 0[0,1.1] | 0[0,-0.900] | 0[0,554.749] | 0[0,-817.050000000001] | 186[139.2,249] | -12[75.6,-12.4] | 1[1,1] | 0[0,0] |
| multiplier | min_0 | 0[0,1] | 0[0,-1] | 0[0,806.75] | 0[0,-565.05] | 270[270,270] | 72[206.4,8.60000] | 1[1,1] | 0[0,0] |
| multiplier | omega_ps_d | 0[0,2] | 0[0,0] | 28.5[0,1526.6] | 28.5[0,154.8] | 241[172.75,270.25] | 43[109.15,8.8500] | 1[1,1] | 0[0,0] |
| multiplier | omega_ps_0 | 0[0,0] | 0[0,-2] | 0[0,0] | 0[0,-1371.8] | NA | NA | NA | NA |
| multiplier | omega_b_d | 0.5[0,1] | 0.5[0,-1] | 57[0,777] | 57[0,-594.8] | 233[189.8,267.2] | 35[126.2,5.800] | 1[1,1] | 0[0,0] |
| multiplier | omega_b_0 | 0[0,1] | 0[0,-1] | 0[0,416.6499] | 0[0,-955.150000000001] | 104.5[41.95,167.05] | -93.5[-21.65,-94.35] | 1[1,1] | 0[0,0] |
| multiplier | theta_d | 0[0,1] | 0[0,-1] | 0[0,850.4] | 0[0,-521.4] | 243[215.1,270.9] | 45[151.5,9.500] | 1[1,1] | 0[0,0] |
| multiplier | theta_0 | 0[0,1.1] | 0[0,-0.900] | 0[0,554.74] | 0[0,-817.050000000001] | 186[139.2,249] | -12[75.6,-12.4] | 1[1,1] | 0[0,0] |
| multiplier | rho_d | 0[0,1] | 0[0,-1] | 0[0,850.4] | 0[0,-521.4] | 243[215.1,270.9] | 45[151.5,9.5000] | 1[1,1] | 0[0,0] |
| multiplier | rho_0 | 0[0,0] | 0[0,-2] | 0[0,0] | 0[0,-1371.8] | NA | NA | NA | NA |
| multiplier | beta_d | 0[0,1.55] | 0[0,-0.450] | 0[0,1343.55] | 0[0,-28.2500000000009] | 238[213.7,274] | 40[150.1,12.6] | 1[1,1] | 0[0,0] |
| multiplier | beta_0 | 0[0,0] | 0[0,-2] | 0[0,0] | 0[0,-1371.8] | NA | NA | NA | NA |
| hatchery | gamma_d | 0[0,0] | 0[0,-1] | 0[0,0.549999] | 0[0,-17.5] | NA | NA | NA | NA |
| hatchery | gamma_0 | 1[0,1] | 1[0,0] | 291[0,3490.15] | 291[0,3472.1] | 3.5[1,282.7] | 1.5[0,278.7] | 285[285,285] | NA |
| hatchery | agedecay_d | 0[0,0] | 0[0,-1] | 0[0,0] | 0[0,-18.05] | NA | NA | NA | NA |
| hatchery | agedecay_0 | 1[1,1] | 1[1,0] | 142046[111451,175690.05] | 142046.5[111451.6,175672] | 1270[6,2729.4] | 1268[5,2725.4] | 3498[3498,3498] | NA |
| hatchery | min_d | 0[0,0.5499] | 0[0,-0.450 | 0[0,9.249999] | 0[0,-8.800000] | 1[1,3.4] | -1[0,-0.600000] | NA | NA |
| hatchery | min_0 | 0[0,1] | 0[0,0] | 0[0,13.85] | 0[0,-4.2] | 1.5[1,7.94999] | -0.5[0,3.949999] | NA | NA |
| hatchery | omega_ps_d | 0[0,1] | 0[0,0] | 0.5[0,15.65] | 0.5[0,-2.4] | 1[1,5.7] | -1[0,1.7] | NA | NA |
| hatchery | omega_ps_0 | 0[0,0] | 0[0,-1] | 0[0,0] | 0[0,-18.05] | NA | NA | NA | NA |
| hatchery | omega_b_d | 0[0,1] | 0[0,0] | 0.5[0,12.3] | 0.5[0,-5.75] | 1[1,3.1] | -1[0,-0.89999] | 12[12,12] | NA |
| hatchery | omega_b_0 | 0[0,0.5499] | 0[0,-0.450] | 0[0,3.7499] | 0[0,-14.3] | 2[2,2] | 0[1,-2] | NA | NA |
| hatchery | theta_d | 0[0,1] | 0[0,0] | 0[0,13] | 0[0,-5.05] | 1[1,6.4] | -1[0,2.4] | NA | NA |
| hatchery | theta_0 | 0[0,0.5499] | 0[0,-0.450] | 0[0,9.2499] | 0[0,-8.8000] | 1[1,3.4] | -1[0,-0.60000] | NA | NA |
| hatchery | rho_d | 0[0,1] | 0[0,0] | 0[0,13] | 0[0,-5.05] | 1[1,6.4] | -1[0,2.4] | NA | NA |
| hatchery | rho_0 | 0[0,0] | 0[0,-1] | 0[0,0] | 0[0,-18.05] | NA | NA | NA | NA |
| hatchery | beta_d | 0[0,1] | 0[0,0] | 0[0,16.85] | 0[0,-1.2] | 1[1,3.2] | -1[0,-0.800000] | NA | NA |
| hatchery | beta_0 | 0[0,0] | 0[0,-1] | 0[0,0] | 0[0,-18.05] | NA | NA | NA | NA |
| broiler | gamma_d | 54.5[49.9,58.55] | -9.5[-4.05,-13.5] | 4128699[3897449,4853409] | -713826[44088.75,-1119129.95] | 53[48.4,54] | 1[0.3999,0] | 1[1,2] | 0[0,-1] |
| broiler | gamma_0 | 72.5[49.8,122.1] | 8.5[-4.15,50.05] | 6020096[4071360,17521295.4] | 1177570.5[217999.95,11548756.25] | 54[50,55] | 2[2,1] | 2[1,4] | 1[0,1] |
| broiler | agedecay_d | 30[22.45,34.55] | -34[-31.5,-37.5] | 28856.5[23678,33396] | -4813669[-3829681.75,-5939142.3] | 53[51,54] | 1[3,0] | 1[1,2] | 0[0,-1] |
| broiler | agedecay_0 | 132[132,132] | 68[78.05,59.95] | 371123761[329729015,394318695] | 366281236[325875654.6,388346156.45] | 40[4,55] | -12[-44,1] | 36[28,42] | 35[27,39] |
| broiler | min_d | 58[55.45,66.55] | -6[1.5,-5.5] | 4955534[4093881,5699845.15] | 113008.5[240520.65,-272694] | 53[48,54] | 1[0,0] | 1[1,3] | 0[0,0] |
| broiler | min_0 | 57.5[46.85,61] | -6.5[-7.100,-11.05] | 4487914.5[3627141.4,4946723] | -354611[-226219,-1025816.1] | 52[49,54] | 0[1,0] | 1[1,2] | 0[0,-1] |
| broiler | omega_ps_d | 60.5[51.35,66] | -3.5[-2.6,-6.05] | 4829238[4163785,5708883] | -13287.5[310425.4,-263655.3] | 53[48.9,54] | 1[0.9000,0] | 1[1,3] | 0[0,0] |
| broiler | omega_ps_0 | 56.5[49.45,61.1] | -7.5[-4.5,-10.95] | 4447061[4080253,4928957] | -395464.5[226892.7,-1043582] | 53[48,54] | 1[0,0] | 1[1,2] | 0[0,-1] |
| broiler | omega_b_d | 87[83.45,92.75] | 23[29.5,20.7] | 8490749[7877223,9099954] | 3648223.5[4023862.65,3127415] | 53[48.8,54] | 1[0.80000,0] | 1[1,3] | 0[0,0] |
| broiler | omega_b_0 | 0[0,0] | -64[-53.95,-72.05] | 0[0,0] | -4842525.5[-3853360.4,-5972539.15] | NA | NA | NA | NA |
| broiler | theta_d | 57[50.9,67] | -7[-3.05,-5.05] | 4960308.5[3984092,5814099.45] | 117783[130731.6,-158439.7] | 52[48,54] | 0[0,0] | 1[1,2] | 0[0,-1] |
| broiler | theta_0 | 58[55.45,66.55] | -6[1.5,-5.5] | 4955534[4093881.05,5699845.15] | 113008.5[240520.65,-272694] | 53[48,54] | 1[0,0] | 1[1,3] | 0[0,0] |
| broiler | rho_d | 57[50.9,67] | -7[-3.05,-5.05] | 4960308.5[3984092,5814099] | 117783[130731.6,-158439.7] | 52[48,54] | 0[0,0] | 1[1,2] | 0[0,-1] |
| broiler | rho_0 | 0[0,0] | -64[-53.95,-72.05] | 0[0,0] | -4842525.5[-3853360.4,-5972539.15] | NA | NA | NA | NA |
| broiler | beta_d | 58[49.45,64.55] | -6[-4.5,-7.5] | 5278657[4538179.2,6455275.25] | 436131.5[684818.8,482736.1] | 52.5[48.85,54] | 0.5[0.85,0] | 1[1,2] | 0[0,-1] |
| broiler | beta_0 | 0[0,0] | -64[-53.95,-72.05] | 0[0,0] | -4842525.5[-3853360.4,-5972539.15] | NA | NA | NA | NA |
